# Bone marrow breakout lesions act as key sites for tumor-immune cell diversification in multiple myeloma

**DOI:** 10.1126/sciimmunol.adp6667

**Published:** 2025-02-07

**Authors:** Raphael Lutz, Alexandra M. Poos, Llorenç Solé-Boldo, Lukas John, Johanna Wagner, Nina Prokoph, Marc A. Baertsch, Dominik Vonficht, Subarna Palit, Alexander Brobeil, Gunhild Mechtersheimer, Nina Hildenbrand, Stefan Hemmer, Simon Steiger, Sabrina Horn, Wojciech Pepke, David M. Spranz, Christoph Rehnitz, Pooja Sant, Jan-Philipp Mallm, Mirco J. Friedrich, Philipp Reichert, Stefanie Huhn, Andreas Trumpp, Karsten Rippe, Laleh Haghverdi, Stefan Fröhling, Carsten Müller-Tidow, Daniel Hübschmann, Hartmut Goldschmidt, Gerald Willimsky, Sandra Sauer, Marc S. Raab, Simon Haas, Niels Weinhold

**Affiliations:** 1 Heidelberg Myeloma Center, Department of Internal Medicine V, Heidelberg University Hospital, Medical Faculty, Heidelberg University, Heidelberg, Germany; 2 Heidelberg Institute for Stem Cell Technology and Experimental Medicine ( HI-STEM), Heidelberg, Germany; 3 Division of Stem Cells and Cancer, German Cancer Research Center ( DKFZ), Heidelberg, Germany; 4 Clinical Cooperation Unit Molecular Hematology/Oncology, Department of Internal Medicine V, Heidelberg University Hospital, Heidelberg University, and German Cancer Research Center ( DKFZ), Heidelberg, Germany; 5 Berlin Institute of Health (BIH) at Charité Universitätsmedizin Berlin, Berlin, Germany; 6 Berlin Institute for Medical Systems Biology, Max Delbrück Center for Molecular Medicine in the Helmholtz Association, Berlin, Germany; 7 Department of Hematology, Oncology and Tumor Immunology, Charité University Medicine, Berlin, Germany; 8 Precision Healthcare University Research Institute, Queen Mary University of London, London, UK; 9 Division of Translational Medical Oncology, German Cancer Research Center ( DKFZ), Heidelberg, Germany; 10 National Center for Tumor Diseases ( NCT), NCT Heidelberg, a partnership between DKFZ and Heidelberg University Hospital, Heidelberg, Germany; 11 Department of Pathology, Heidelberg University Hospital, Medical Faculty, Heidelberg University, Heidelberg, Germany; 12 Tissue Bank of the National Center for Tumor Diseases ( NCT) Heidelberg, Germany; 13 Department of Orthopaedics, Heidelberg University Hospital, Medical Faculty, Heidelberg University, Heidelberg, Germany; 14 Division of Chromatin Networks, German Cancer Research Center ( DKFZ) and BioQuant, Heidelberg, Germany; 15 Institute of Immunology, Charité-Universitätsmedizin Berlin, corporate member of Freie Universität Berlin and Humboldt-Universität zu Berlin, Berlin, Germany; 16 Department of Radiology, Heidelberg University Hospital, Medical Faculty, Heidelberg University, Heidelberg, Germany; 17 Single Cell Open Lab, German Cancer Research Center ( DKFZ), Heidelberg, Germany; 18 German Cancer Consortium ( DKTK), Heidelberg, Germany; 19 Institute of Human Genetics, Heidelberg University, Heidelberg, Germany; 20 Department of Internal Medicine V, Heidelberg University Hospital, Medical Faculty, Heidelberg University, Heidelberg, Germany; 21 Computational Oncology, Molecular Precision Oncology Program, National Center for Tumor Diseases ( NCT) Heidelberg and German Cancer Research Center ( DKFZ), Heidelberg, Germany; 22 GMMG-Studygroup at Heidelberg University Hospital, Department of Internal Medicine V, Heidelberg University Hospital, Medical Faculty, Heidelberg University, Heidelberg, Germany; 23 German Cancer Consortium ( DKTK), partner site Berlin, Berlin, Germany; 24 German Cancer Research Center ( DKFZ), Heidelberg, Germany

## Abstract

The bone marrow microenvironment plays a crucial role in the development of multiple myeloma. As the disease progresses, malignant myeloma cells can evolve to survive outside the bone marrow. However, the processes underlying bone marrow independence and their consequences for immune control remain poorly understood. Here, we conducted single-cell and spatial multi-omics analyses of bone marrow-confined intramedullary disease and paired breakout lesions that disrupt the cortical bone. These analyses revealed a distinct cellular microenvironment and architectural features of breakout lesions, characterized by extensive areas of malignant plasma cells interspersed with lesion-specific solitary NK and macrophage populations, as well as focal accumulations of immune cell agglomerates. Within these agglomerates, spatially confined T cell clones expanded alongside various immune cells, coinciding with the local genomic evolution of tumor cells. These analyses identify breakout lesions as a hotspot for tumor-immune cell interactions and diversification, representing a key event in myeloma pathogenesis.

## Introduction

Multiple myeloma (MM) is a monoclonal plasma cell malignancy that remains fatal for the majority of patients despite new treatment options (1). Typically, malignant plasma cells expand almost exclusively in the bone marrow (BM) (2). In the precursor stages of MM, diffuse BM infiltration is the predominant growth pattern of malignant cells (3, 4). In contrast, the formation of nodular accumulations of tumor cells, known as focal lesions, is closely correlated with the onset of symptomatic disease and is observed in most MM patients (3–6). As the disease progresses, MM cells from focal lesions may disrupt the cortical bone and grow as soft tissue masses adjacent to the bone, also known as breakout lesion or paramedullary disease. An even more advanced stage is extramedullary disease (EMD), in which the malignant tissue expands outside of the skeletal system (7). In patients treated with novel immunotherapies, the presence of EMD has emerged as one of the most important prognostic markers (8), highlighting the loss of BM dependence as a feature of advanced, aggressive disease. However, BM-independent MM cells from end-stage patients usually fail to expand *in vitro*, suggesting a persistent dependence on interactions with cellular and non-cellular components of the tumor microenvironment (TME) (9).

Data from preclinical models support a critical role of the TME in extramedullary spread (10). However, the early processes associated with BM independence in human disease and its implications for immune control remain poorly understood. With the hypothesis that focal lesions represent an intermediate stage towards EMD, we recently performed single-cell sequencing of paired samples from diffusely infiltrated random BM (rBM) sites in the pelvis and intramedullary (BM-confined) lesions (11). Interestingly, although modest tumor load-dependent changes in the tumor microenvironment (TME) were noted, no site-specific immune responses were detected in intramedullary focal lesions, even though the MM cells in these lesions exhibited distinct mutational profiles. A possible explanation may be that intramedullary lesions are still dependent on the BM niche and directly exposed to its unique regulatory immune environment, dominated by immature immune and memory cells (12, 13). In contrast, MM soft tissue masses arising from bone lesions that disrupt the cortical bone, which are observed in 15-20% of newly diagnosed MM (NDMM) patients, appear to be able to survive outside the BM and may be less exposed to the immunoregulatory BM niche (7, 14). However, the cellular and spatial architecture of these breakout lesions, their function in tumor immunology, and their overall role in MM pathogenesis remain poorly understood.

To elucidate the early processes associated with BM independence, we dissected the cellular, immunological, and spatial ecosystem of breakout lesions from NDMM patients. Our results show that breakout lesions are sites of bidirectional tumor-immune interactions. They consist of extensive areas dominated by MM cells and largely devoid of immune cells, with the exception of interspersed macrophages and NK cells with distinct transcriptional profiles. Focal accumulations of immune cells were observed within the tumor-dominated areas, typically associated with blood vessels. Such ‘immune islands’ are active sites for tumor T cell interactions, leading to site-enriched clonal T cell expansion and potential immune exhaustion. Collectively, these data highlight the importance of breakout lesions as key sites for tumor-immune interactions, with implications for understanding the processes underlying tumor cell dissemination and therapeutic strategies in the treatment of MM.

## Results

### Multiple myeloma breakout lesions harbor a distinct cellular ecosystem

**Fig 1:**
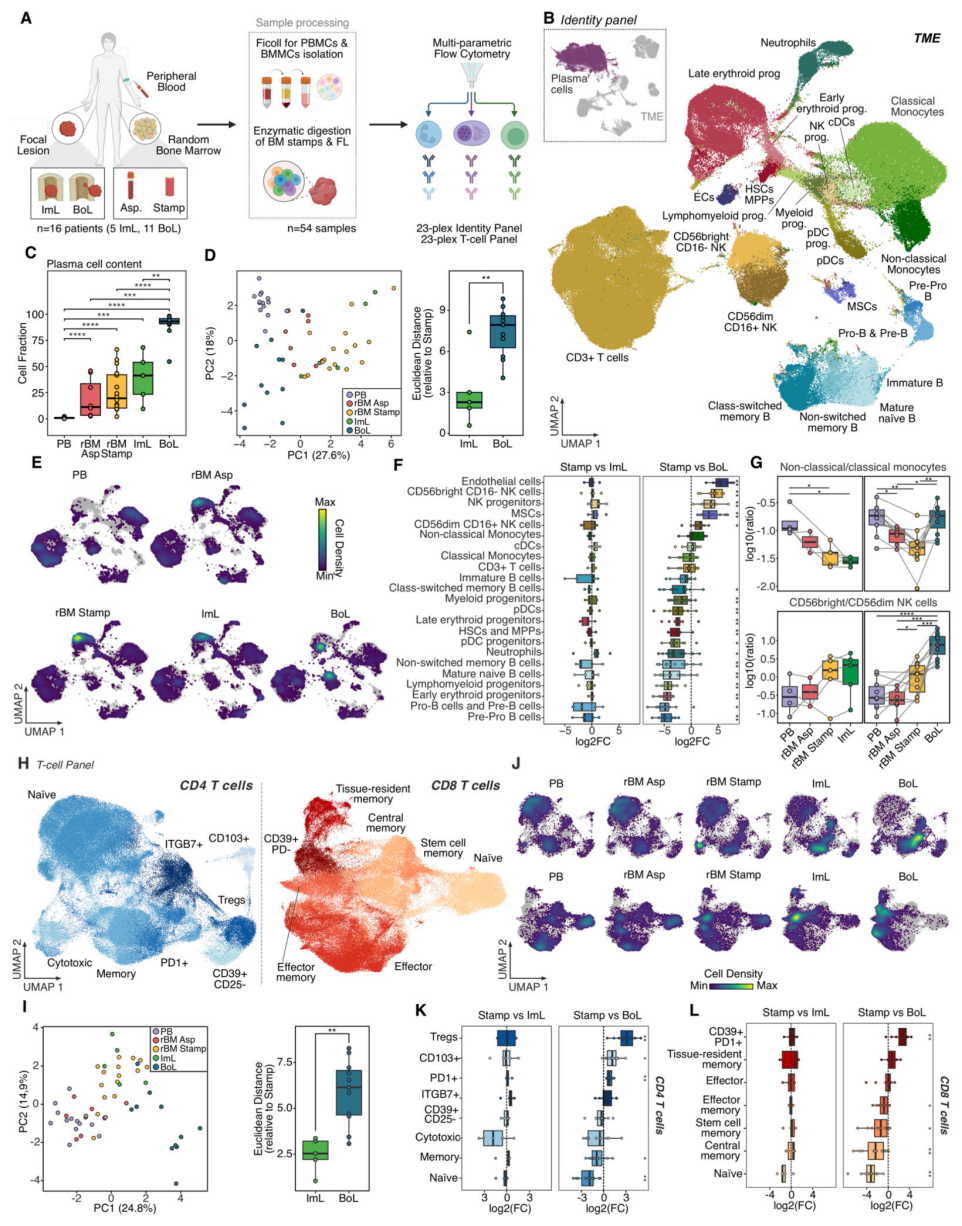
The cellular ecosystem of focal lesions and paired random bone marrow. A) Experimental design. 54 matched samples, including 14 peripheral blood (PB), 8 random bone marrow (rBM) aspirates, 16 rBM trephine biopsies (stamps), 5 intramedullary lesions (ImL), and 11 breakout lesions (BoL) from 16 newly diagnosed multiple myeloma (NDMM) patients were analyzed using two 23-plex cytometry panels. PBMC/BMMC: PB or BM mononuclear cells. B) UMAP representation of the identity panel (top UMAP, n=150,000 sketched cells) including cells from PB, rBM aspirates and stamps, ImL and BoL. In the bottom UMAP, the UMAP representation was re-calculated after plasma cell exclusion (n=300,000 sketched cells). C) Plasma cell content of samples. D) Left: Principal Component Analysis (PCA) based on cell type fractions, excluding plasma cells. Right: Euclidean distance of ImL and BoL to respective paired rBM. E) UMAP depicting cellular density after exclusion of plasma cells across all sample types (n=10,000 projected cells per sample type). F) Relative cell type abundance in ImL and BoL. Data is represented as log2(fold-change) compared to average cell type abundance in paired rBM stamps. G) Cell fraction ratios between non-classical and classical monocytes (upper panel) and CD56bright and CD56dim NK cells (lower panel) across all sample types. Patients are separated by focal lesion type. H) UMAP representation of CD4 (left; n=150,000 sketched cells) and CD8 (right; n=138,301 sketched cells) T cells based on the T cell panel including cells from PB, rBM aspirates and stamps, ImL and BoL. I) Left: PCA based on cell type fractions from all identified T cell subtypes. Right: Euclidean distance of ImL and BoL to respective paired rBM stamps. J) UMAP depicting cellular density for CD4 (upper panel; n=6,000 projected cells per sample type) and CD8 (lower panel; n=7,000 projected cells per sample type) T cells across all sample types included in the study. K+L) Differential abundant CD4 (K) and CD8 (L) subtypes between ImL or BoL and their respective rBM stamps. Data is represented as log2(fold-change) compared to average cell type abundance in respective paired rBM stamps. Statistical analyses were performed using Wilcoxon tests, unpaired in (C, D, G and I) and paired in (F, G, K, L). *P <0.05, **P <0.01, ***P <0.001, ****P<0.0001.

To systematically elucidate the cellular composition of MM breakout lesions, we first employed a data-driven 23-plex cytometry approach (15), designed to quantitatively map all major BM-resident cell types (“identity panel”; see Methods, Table S1). Using this approach, we analyzed enzymatically digested breakout lesions and BM-confined intramedullary lesions from 11 and 5 NDMM patients, respectively (Fig. 1A, Fig. S1A-B and Data file S1). To enable comparative analyses, we included paired samples of digested trephine biopsies (rBM stamps, n=16), liquid aspirates from diffusely infiltrated rBM sites at the iliac crest (rBM Asp, n=8), and peripheral blood (PB, n=14) (Data file S2). After batch correction, data integration and cell type annotation, we obtained a quantitative representation of 7,609,355 high-quality cells across all samples, representing 54 ecosystems (Fig. 1B and Fig. S1C-G). These encompassed 24 cell types, including various B cell, T cell, natural killer (NK) cell, myeloid, plasma cell, hematopoietic stem and progenitor, stromal and endothelial cell subsets (Data file S3).

As expected, plasma cell levels gradually increased from PB to rBM and BM-confined intramedullary lesions, peaking in breakout lesions with a mean infiltration of 89.8% (range: 54.7-98.2%) (Fig. 1C). Compared to rBM, the B-cell lineage markers CD19 and CD27 were significantly downregulated in plasma cells from breakout lesions, whereas CD16 and the adhesion molecule CD56 were upregulated (Fig. S2A-B), suggesting an adapted immunophenotype of the tumor cells in breakout lesions. In contrast, plasma cells from intramedullary lesions did not display differences to their rBM counterparts (Fig. S2A-B). In line with this, whole genome sequencing (WGS) revealed a more pronounced degree of spatial subclonal heterogeneity in breakout lesions compared to intramedullary lesions, relative to their corresponding rBM pairs (p=0.04, Fig. S2C-D). However, no consistent differences in cytogenetic risk were identified between breakout lesions, intramedullary lesions, and their respective paired rBM (Data file S1).

To investigate the TME in detail, we excluded plasma cells from further analysis. Principal component analysis (PCA) considering cellular compositions revealed that breakout lesions harbored a TME distinct from their paired rBM samples and intramedullary lesions (Fig. 1D, Fig. S3). In line with this, major cellular shifts were observed in a variety of cell types (Fig. 1E-G). For example, breakout lesions contained considerably higher numbers of mesenchymal stromal cells and endothelial cells when compared to rBM samples, suggesting a stromal environment with high vascularization (Fig. 1E-F). In contrast, stem and progenitor populations were largely underrepresented in breakout lesions compared to all types of BM samples, indicating that these regions play a less active role in hematopoiesis. Notably, the TME of breakout lesions was highly enriched in NK cells, which displayed a strong shift towards an inflammatory CD56bright phenotype (Fig. 1G). Similarly, monocyte subsets showed an adopted phenotype, with a major shift towards CD16 expression (Fig. 1G).

Next, we investigated the above-mentioned samples using a 23-plex T cell panel, designed to explore the T cell landscape in detail (Table S2). This uncovered 16 T cell subsets across 1,352,284 high-quality T cells, including CD4 and CD8 naïve, memory, effector, and tissue-resident T cells, regulatory T cells (Tregs), and gamma-delta (gd) T cells (Fig. 1H and Fig. S4). PCA analysis demonstrated a distinct T cell landscape in breakout lesions compared to intramedullary lesions, rBM and PB (Fig. 1I, Fig. S3B). In particular, breakout lesions were depleted of CD4 and CD8 naïve and memory T cells and showed a strong enrichment of PD1+ CD4 T cells, tissue-resident CD4 T cells, CD38+ gd T cells, and Tregs (Fig. 1J-L and Fig. S4E). Most notably, a CD8 T cell cluster characterized by high expression of the exhaustion and checkpoint molecules CD39 and PD1 was almost exclusively detectable in breakout lesions.

Together, these data demonstrate that breakout lesions harbor a cellular ecosystem that is distinct from intramedullary focal lesions and from diffusively infiltrated rBM.

### Altered niche in breakout lesions confers reduced hematopoiesis-supporting capacity and active vascularization

**Fig 2:**
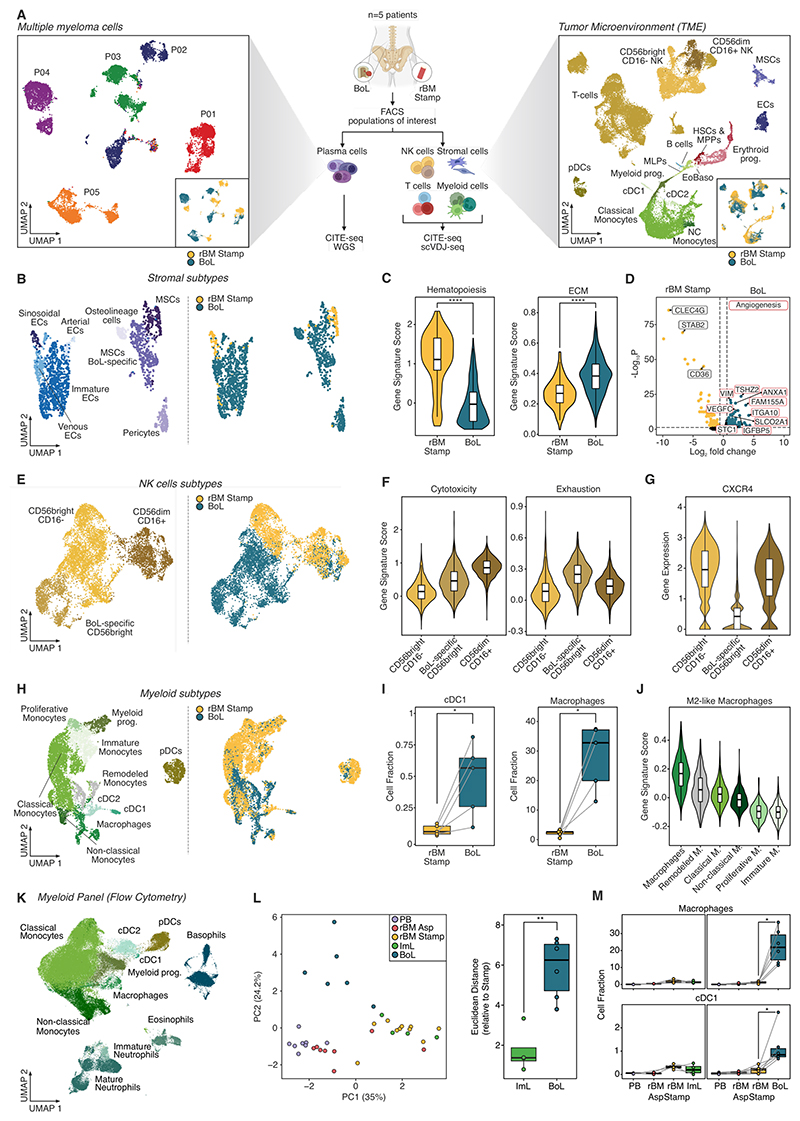
Cell states in breakout lesions and paired random bone marrow. A) Experimental design for the in-depth characterization of breakout lesions and paired random bone marrow stamps from 5 patients. Cell types of interest, including plasma, NK, myeloid, mesenchymal stromal, endothelial and T cells were sorted and analyzed using single-cell proteo-genomics (CITE-seq) and VDJ-based T cell receptor (TCR) sequencing. Plasma cells were also analyzed using whole genome sequencing (WGS). Left: UMAP representation of the plasma cell CITE-seq data. Each cell is colored by patient and sample location (BoL or rBM). Right: UMAP representation of CITE-seq data for the tumor microenvironment cells. Each cell is colored by the annotated cell type and sample location (BoL or rBM). EoBaso: eosinophil/basophil/mast cell progenitors; MLPs: lymphomyeloid progenitors; NC monocytes: non-classical monocytes. B) UMAP representation of CITE-seq data for stromal and endothelial cells. Cells are colored by subtype (left) and sample location (right). C) Violin and box-whisker plots showing the expression of hematopoiesis-supporting factors (left) and extra-cellular matrix production (right) in BoL and rBM stamps. D) Volcano plot for the comparison of gene expression of endothelial cells in BoL and rBM stamps. Significantly differentially expressed genes are depicted in yellow or blue (Wilcoxon rank sum test, Benjamini Hochberg adjusted P<0.05, ≥1.5-fold enrichment). Genes associated with angiogenesis are highlighted in red. E) UMAP representation of CITE-seq data for NK cells. Cells are colored by subtype (left) and sample location (right). F) Violin and box-whisker plots displaying cytotoxicity (24) and exhaustion (23) gene signatures for indicated NK cell subtypes. G) Violin and box-whisker plots showing CXCR4 expression for the indicated NK cell subtypes. H) UMAP representation of CITE-seq data for myeloid cells. Cells are colored by subtype (left) and sample location (right). I) Cell fraction of conventional dendritic cells 1 (cDC1) (left) and macrophages (right) across the 5 patients with paired BoL and rBM stamps. Fractions were calculated considering only the myeloid compartment. J) Violin and box-whisker plots showing the gene signature for an M2-like macrophage phenotype (25) for the myeloid subtypes. K) UMAP representation of flow cytometry data for myeloid cells (n=96,031 sketched cells). The data was collected for PB, rBM aspirates and stamps as well as ImL and BoL samples from 10 patients using a designated myeloid panel. L) Left: Principal Component Analysis (PCA) based on cell type fractions from all identified myeloid cell subtypes. Right: Euclidean distance of ImL and BoL lesions to respective paired rBM stamps. M) Cell fractions of macrophages (upper panel) and cDC1 cells (lower panel) across all sample types included in the study. Statistical analyses in (C), (I) and (L-M) were performed using the Wilcoxon test. In (I) and (M) ImL or BoL with paired rBM stamps were compared. *P <0.05, **P <0.01, ***P <0.001, ****P<0.0001.

To further elucidate cellular features of resident cells in breakout lesions, we performed single-cell proteo-genomics (CITE-seq) of flow-enriched cell types of interest, such as plasma cells, myeloid, NK, and T cells, as well as mesenchymal stromal and endothelial cells from 5 breakout lesions and matched rBM samples, resulting in 13,057 plasma cells and 39,552 high-quality TME cells (Fig. 2A, Fig. S5, Table S3 and Data file S3).

Breakout lesions develop from BM-confined lesions by disruption of the cortical bone, followed by the outgrowth of malignant cells in soft tissue masses. Here, we investigated the potential role of the non-hematopoietic niche in this process (Fig. 2B-D). As expected, rBM stromal cells were characterized by leptin receptor (LEPR) expression and high production of hematopoiesis-supporting factors such as *CXCL12, KITL* and *IL7 (16)*. In breakout lesions, the majority of stromal cells also expressed *LEPR*, but displayed a reduced production of hematopoiesis-supporting factors (Fig. 2C and Fig. S6A), consistent with the impaired hematopoiesis in breakout lesions as quantified by flow cytometry (compare Fig. 1). Moreover, stromal cells in breakout lesions showed signs of increased differentiation toward the osteoblastic lineage (e.g., *RUNX2, SP7*) (17) and elevated levels of extracellular matrix production (e.g., *COL3A1, SPARC*) (Fig. 2C and Fig. S6A).

Endothelial cells in the rBM predominantly displayed a *STAB2*-positive sinusoidal phenotype (Fig. 2D), in line with previous reports (16). In contrast, endothelial cells in breakout lesions were *STAB2*-negative, displayed phenotypes associated with arterial (e.g. *HEY1, SOX17*), venous (e.g., *SELP, ACKR1*) or immature (e.g., *IGFBP5, TSHZ2*) identities (18), and were characterized by the expression of genes associated with tumor angiogenesis and endothelial sprouting (e.g. *ANXA1, STC1*) (19–21) (Fig. 2B,D and Fig. S6B), suggesting ongoing neovascularization. Interestingly, arterial endothelial cells in breakout lesions produced elevated levels of *CXCL12*, potentially mediating recruitment of immune cells to these sites (22).

Importantly, while the described characteristic features of breakout lesion-resident stromal and endothelial cells were observed across patients, considerable inter-patient heterogeneity with regards to the expression of specific gene programs was observed, likely driven by patient-specific tumor-TME interactions (Fig. S6C and Data file S3). In line with this, we observed a considerable site-specific modulation of transcriptomic states of malignant plasma cells across all patients (Fig. 2A), which was consistent with pronounced differences in the subclonal architecture determined by WGS (Fig. S2D).

Taken together, these results suggest an enrichment of stromal cells in breakout lesions that may contribute to the formation of soft tissue masses extruding from the bone, but have a reduced capacity to support hematopoiesis. In addition, breakout lesion endothelial cells adopt phenotypes distinct from the BM and likely contribute to neovascularization.

### Breakout lesions are primary sites for tumor-immune cell interactions

Next, we employed our CITE-seq data to characterize the transcriptomic and immunological states acquired by NK and myeloid cells in breakout lesions. These cells displayed significant and concordant transcriptomic alterations when compared to their respective counterparts in the rBM, indicative of sustained tumor-immune interactions (Fig. 2A). In line with our cytometric analysis, we observed a remodeling of NK cells in the TME of breakout lesions, including an enrichment of CD56bright NK subsets (Fig. 2E and Fig. S6D-E). Notably, a specific NK cell subset, characterized by a particularly high expression of CD56, was exclusively identified in breakout lesions (Fig. 2E and Fig. S6F). Breakout lesion-specific NK cells displayed an intermediate expression of cytotoxicity-related genes, distinct transcriptional profiles for inhibitory/activating receptors, strong expression of genes associated with immunological exhaustion and reduced *CXCR4* levels (Fig. 2F-G and Fig. S6D-E), phenocopying previously described changes of NK cells in solid tumors (23, 24) and suggestive of their pronounced interaction with myeloma cells.

Similar to the NK cell compartment, major alterations in the cellular composition and transcriptional states of the myeloid compartment were observed in breakout lesions. Consistent with flow cytometry, the ratio of non-classical to classical monocytes was significantly increased in breakout lesions (Fig. S6J). Due to the higher resolution of CITE-seq compared to cytometry, we observed additional changes in the cellular composition, including an enrichment of conventional dendritic cells 1 (cDC1) and macrophages in the TME of breakout lesions (Fig. 2I and Fig. S6J). Importantly, macrophages in breakout lesions resembled well described tumor-associated macrophages in solid tumors (TAMs) and were characterized by an M2-like phenotype (25) (Fig. 2J and Fig. S6G-H).

To further validate these changes in the cellular composition of the myeloid compartment, we performed cytometric ecotyping using a 23-plex myeloid-focused panel across paired samples from 10 patients, including PB, rBM (stamps and aspirates), and both breakout and intramedullary lesions (Fig. 2K-M, Fig. S7, Table S4 and Data file S2). This uncovered 11 subsets across 703,084 high-quality cells from 36 matched samples (Fig. 2K and Fig. S7A-C). Notably, this analysis revealed a distinct composition of the myeloid compartment in breakout lesions when compared to matched rBM and intramedullary lesions (Fig. 2L and Fig. S3C), and confirmed all previous findings, including the enrichment of cDC1 and macrophages in breakout lesions (Fig. 2M and Fig. S7D-F). Jointly, these data reveal a distinct immunological state of NK and myeloid cells in myeloma breakout lesions, indicative of persistent tumor-immune cell interactions.

### Breakout lesions harbor a distinct T cell repertoire dominated by clonally-expanded T cells

**Fig 3:**
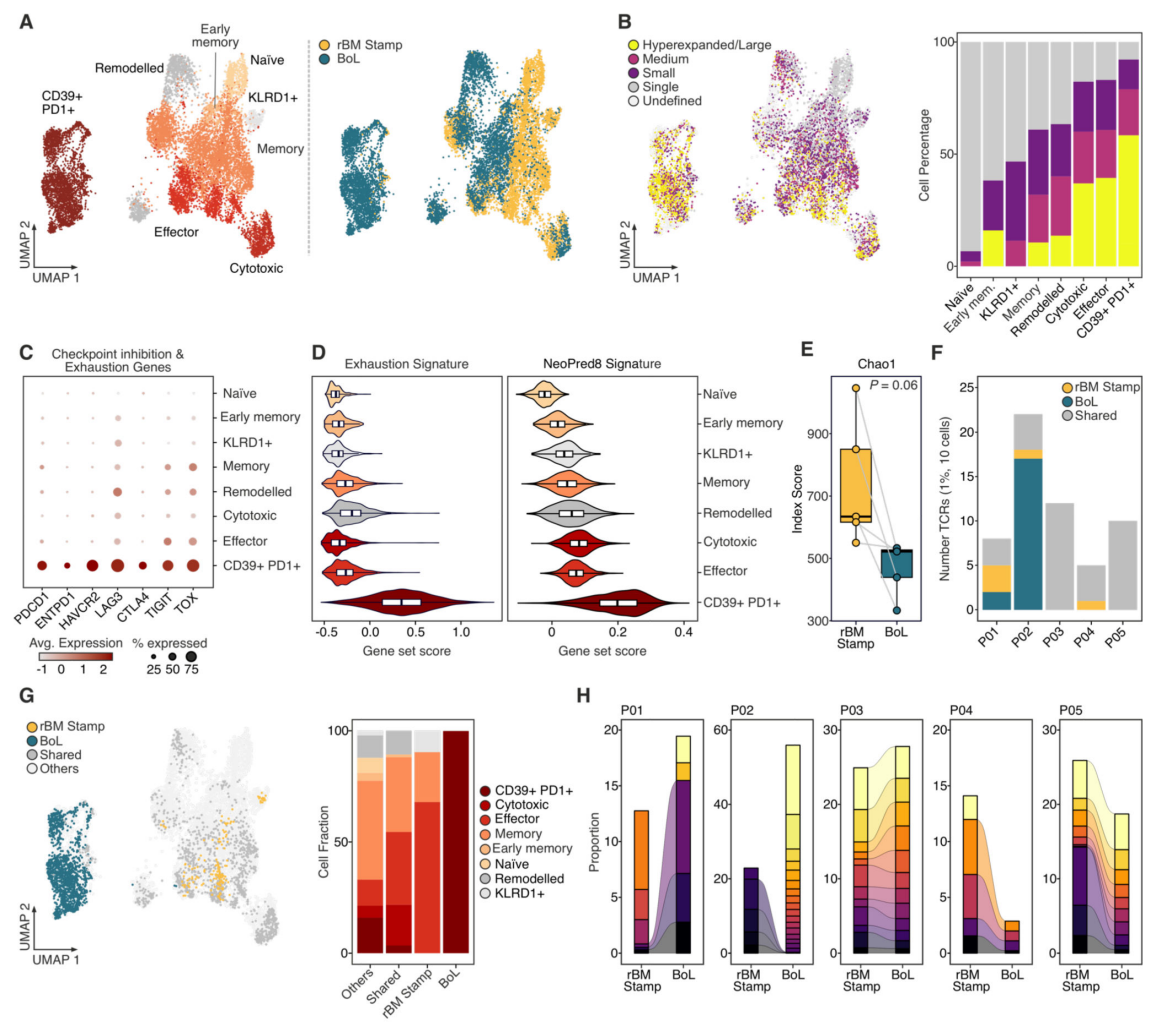
T cell repertoire of breakout lesions and paired random bone marrow. A) UMAP representation of CITE-seq data for CD8 T cells from 5 patients with paired samples. Cells are colored by cell type (left) and sample location (right). B) Left: the same UMAP as in a) colored by the level of T cell expansion. Undefined: T cells without T cell receptor (TCR) information. Right: Proportion of expanded T cells per CD8 T cell subtype. T cells without TCR information were excluded. C) Expression of checkpoint inhibitor molecules per CD8 T cell subtype. Color and size of points indicate the average expression and the percentage of positive cells, respectively. D) Violin and box-whisker plots for a T cell exhaustion signature (28) and a signature of neoantigen-reactive tumor-infiltrating lymphocytes from solid cancers (27) per CD8 T cell subtype. E) T cell diversity according to the Chao1 index for BoL and paired rBM stamps. The boxplots show the median and the interquartile range, while the upper and lower whiskers show the highest and lowest value (excluding outliers), respectively. The p value was calculated using the Wilcoxon signed rank test. F) Total number of T cell clones that were shared between BoL and rBM stamp (gray), or unique to either the rBM stamp (yellow) or the BoL (blue). Only T cell clones with a proportion of ≥1% and ≥10 cells in at least one of the paired samples were considered. G) T cell clones that were unique to BoL (blue) or rBM stamps (yellow) and shared T cell clones are highlighted in the UMAP for CD8 T cells (left) and assigned to CD8 T cell subtypes (right). CD8 T cell clones with a proportion <1% and/or <10 cells in both of the paired samples were classified as “Others”. H) CD8 T cell proportion of all expanded T cell clones in paired BoL and rBM stamps, considering clones with a proportion of ≥1% and ≥10 cells in at least one of the paired samples.

Our flow cytometry studies uncovered dramatic changes within the T cell compartment of breakout lesions, including a strong enrichment of CD39+/PD1+ CD8 T cells (Fig. 1). CITE-seq combined with single-cell T cell receptor (TCR) sequencing revealed that breakout lesion-resident CD39+/PD1+ CD8 T cells were predominantly expanded T cell clones, overexpressing a variety of additional checkpoint inhibition and exhaustion molecules, including *CTLA4, LAG3*, Tim3 (*HAVCR2*), *TIGIT* and *TOX* (Fig. 3A-D). Thus, breakout lesion-resident CD39+/PD1+ CD8 T cells appear to be antigen-experienced and potentially exhausted (26). The majority of expanded clones in breakout lesions did not match previously described TCRs associated with pathogen-related T cell responses, suggesting that CD39+/PD1+ CD8 T cells are likely myeloma-specific (Fig. S6K and Data file S4). In line with this, CD39+/PD1+ CD8 T cells highly overexpressed a gene signature representative of neoantigen-reactive tumor-infiltrating lymphocytes in solid cancers (27) (Fig. 3D).

Consistent with the expansion of T cell clones and the resulting lower T cell receptor diversity, a reduced Chao1 index was observed in T cell receptor repertoires of breakout lesions when compared to their paired rBM counterparts (Fig. 3E). Two patients (P01 and P02) showed a particularly strong enrichment of CD39+/PD1+ CD8 T cells in their breakout lesions (Fig. S6L). Notably, the vast majority of these cells were expanded T cell clones that were not detectable in the paired rBM from the same patient (Fig. 3F-H and Data file S4), suggesting site-enriched T cell responses, further highlighting breakout lesions as a key site for both tumor-immune interactions.

### Spatially-resolved single-cell analysis reveals microregional tumor-immune interactions

**Fig 4:**
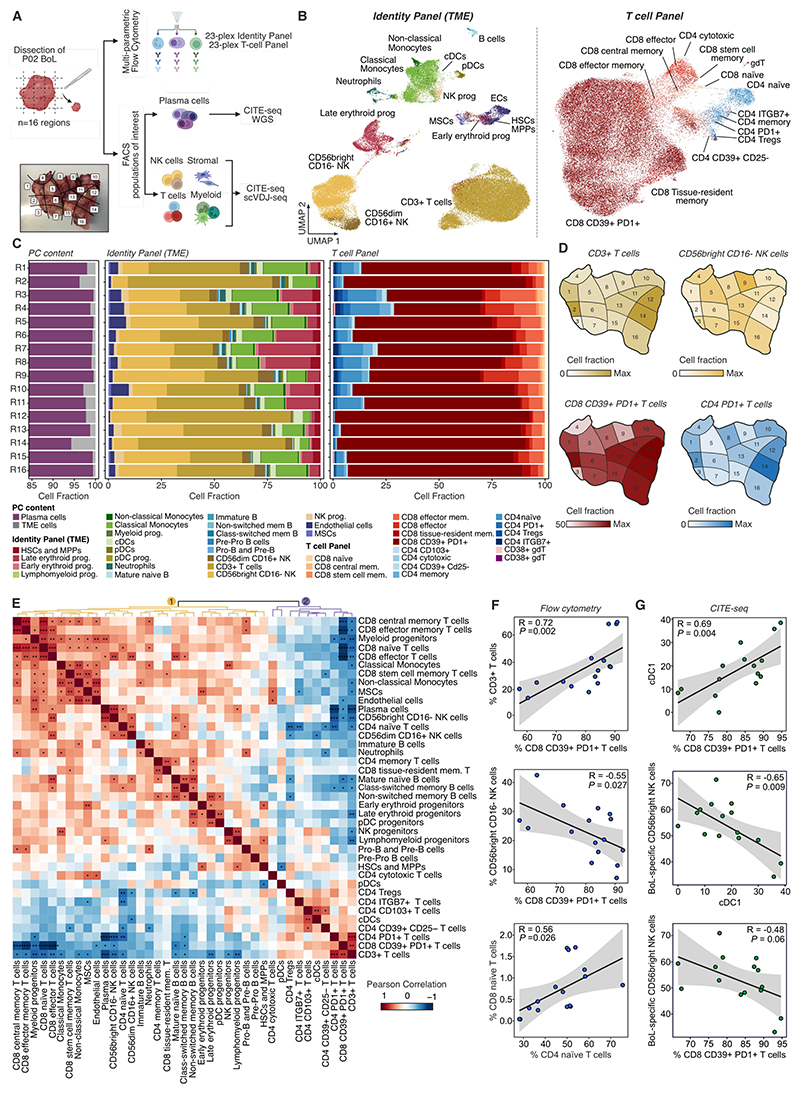
Spatially-resolved cellular ecotyping of a breakout lesion. A) Experimental design for the analysis of 16 spatially-resolved regions of the breakout lesion of patient P02 using flow cytometry, single-cell proteo-genomics (CITE-seq) and VDJ-based T cell receptor (TCR) sequencing. B) Left: UMAP representation of flow cytometry data collected using the identity panel including all cells from the 16 regions except for plasma cells (n=63,671 cells). Right: UMAP representation of T cells analyzed with the T cell panel including all T cells from the 16 regions (n=46,442 cells). C) Bar plots depicting cellular fractions for the 16 regions obtained using the identity panel (Left: plasma cell count; Middle: TME cell fractions) as well as the T cell panel (Right). D) Spatial distribution of selected cell types across the 16 regions. Regions are colored based on relative fractions for TME cell types (upper panel) or relative fractions from CD4 and CD8 T cell subsets (lower panel). E) Correlation matrix of cell fractions obtained from flow cytometry data. Plasma cell counts, relative fractions for TME cell types (identity panel) and relative fractions from CD4 and CD8 T cell subsets (T cell panel) were used. Color represents Pearson’s correlation coefficient values. Statistically significant correlations are highlighted: *P <0.05, **P <0.01, ***P <0.001, ****P<0.0001. F) Examples of statistically significant correlations between cell fractions from flow cytometry in the 16 regions depicted as scatter plots. G) Correlations between CD39+/PD1+ CD8 T cells, cDC1 and BoL-specific CD56bright NK cells based on CITE-seq data for 15/16 regions of patient P02 depicted as scatter plots. For the calculation of cell fractions only cells from the same cellular compartment were considered to account for the cell sorting prior to CITE-seq.

To further investigate subregion-specific tumor immune-interactions, we dissected a particularly large breakout lesion from patient P02 into 16 spatially defined microregions and performed cytometric cellular ecotyping, yielding a total of 63,671 TME cells (identity panel) and 46,442 T cells (T cell panel), and CITE-seq with paired TCR sequencing, yielding 16,462 TME cells, across individual regions (Fig. 4A and Fig. S8A-B). First, using our spatially-resolved cytometry data, we determined plasma cell levels, quantified cellular abundances of the TME and mapped T cell subsets across all regions (Fig. 4B). While all subregions showed MM cell infiltration levels >90%, this analysis uncovered significant spatial variation in the absolute and relative composition of immune cells, with CD39+/PD1+ CD8 T and CD56bright NK cells being most regionally variable, suggesting differences in local hotspots of tumor-immune interactions (Fig. 4C-D and Fig. S8A).

To investigate co-localization of immune cell types within such hotspots, we performed a correlation analysis of cellular abundances across the regions (Fig. 4E and Data file S5). This revealed two major clusters, consisting of cell types quantitatively coinciding in spatial regions. Cluster 2 was predominated by immunoregulatory Tregs and a group of T cell subtypes with an antigen-experienced phenotype, including PD1 or CD39 expressing CD4 T cells, and the above described clonally expanded CD39+/PD1+ CD8 T cells, indicating a concerted immunoregulatory or antigen-experienced environment (Fig. 4E). The abundance of cluster 2 cell types generally correlated with the total amount of T cells and cDC1s, suggesting the establishment of local immunoregulatory T cell environments as a consequence of the expansion of antigen-experienced T cells (Fig. 4E-G). In contrast, cluster 1 was associated with high plasma cell levels and the absence of T cells with an antigen-experienced phenotype. In line with this, the presence of clonally expanded CD39+/PD1+ CD8 T cells was anti-correlated with plasma cell counts and breakout lesion-specific NK cell subsets, indicating their spatial separation (Fig. 4E-G).

Spatially resolved cytometry analyses from six additional patients confirmed the high degree of intra-lesion heterogeneity within the immune compartment of breakout lesions (Fig. S8C and Data file S5). For one patient with high (P11) and one patient with low (P05) levels of CD39+/PD1+ CD8 T cells, sufficient regions were available for correlation analysis. While the patient with high levels of CD39+/PD1+ CD8 T cells (P11) showed features very similar to those of patient P02, less overall spatial heterogeneity was observed in the patient with low number of antigen-experienced T cells (P05) (Fig. S8D-E).

Together, these analyses revealed significant regional heterogeneity in the distinct immune cell communities within breakout lesions, linked to the presence of T cells with an antigen-experienced phenotype.

### Spatially-separated co-evolution of genomic tumor cell diversification and expanded T cell clones

**Fig 5:**
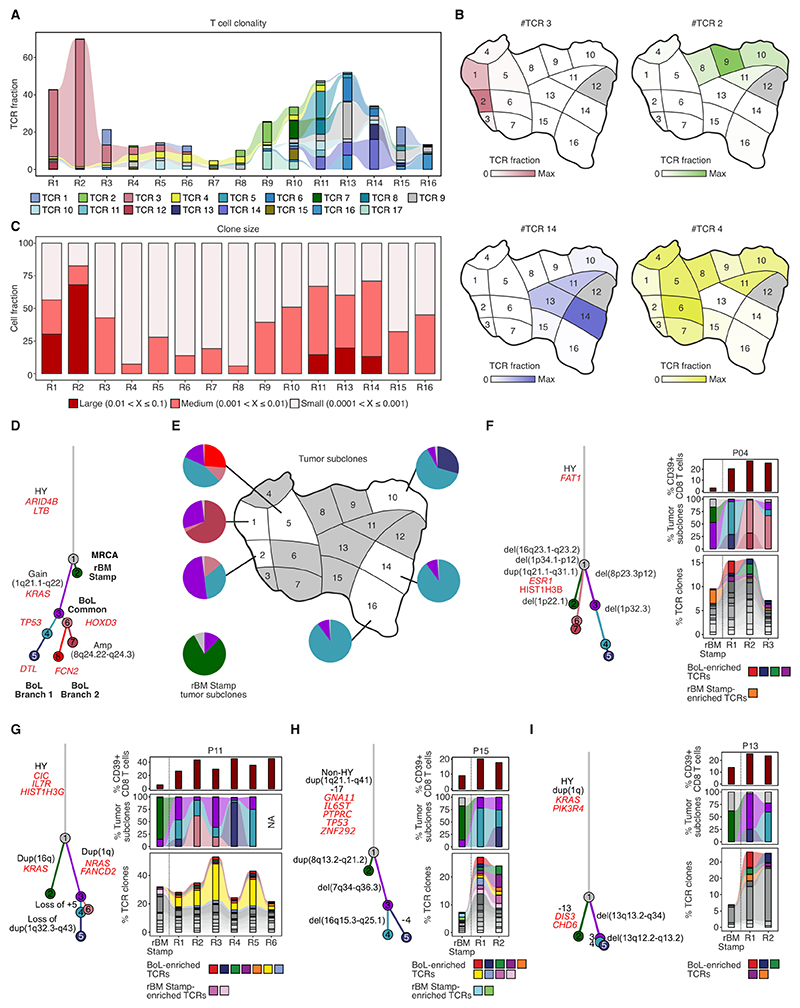
Spatial distribution of T cell clones and tumor subclones within breakout lesions. A) Distribution of expanded T cell clones across the breakout lesion (BoL) of patient P02 according to single-cell T cell receptor (TCR) sequencing. We considered only T cell clones with at least 25 cells in at least one region which were not detectable in the paired random bone marrow (rBM) stamp and peripheral blood. Due to insufficient sample material, CITE- and TCR-seq data was not available for region 12. B) Spatial distribution of four selected T cell clones across the regions. Regions are colored according to the proportion of the indicated T cell clone. C) Total proportion of expanded T cell clones per region. D) Phylogenetic tree based on whole genome sequencing (WGS) data for 6 selected regions from the BoL (R1, R2, R5, R10, R14 and R16) and the paired rBM stamp of patient P02. Selected mutations and copy number aberrations are highlighted in red and black, respectively. HY: hyperdiploid karyotype, MRCA: most recent common ancestor. E) Pie charts showing the proportion of the identified tumor subclones per region. The subclones are color coded in line with the phylogenetic tree in (D). F-I) Bulk WGS and TCR data for four additional patients with multiple regions per breakout lesion. Left panel: Phylogenetic tree. dup: chromosomal duplication; del: chromosomal deletion. Upper right panel: Barplots depicting the relative cell fraction of CD39+/PD1+ CD8 T cells per sample as determined by flow cytometry. Middle right panel: Alluvial plot depicting the fraction of tumor subclones per region. Bottom right panel: Alluvial plot depicting cell fractions of expanded TCR clones (≥1% of total TCR repertoire) per region. Shared TCRs are shown in shades of gray, while enriched TCR clones (fold-change ≥ 10) are depicted in color.

To investigate potential causes underlying intra-lesion heterogeneity, we analyzed single-cell TCR sequencing data of the spatially separated microregions of patient P02. This analysis revealed that distinct lesion-specific T cell clones were operational in distinct regions within the lesion (Fig. 5A-C, Fig. S9A-B and Data file S6). For example, the hyper-expanded T cell clone #3 dominated the adjacent regions R1 and R2, but was not observed in the distally located regions R8-R16 (Fig. 5A-B and Fig. S9B). Conversely, these regions harbored several expanded T cell clones (i.e., TCR clone #2 and clone #14) that were not present in regions R1 and R2 (Fig. 5A-C and Fig. S9B).

To address the question whether the spatial heterogeneity of clonal T cell responses might be a consequence of divergent genomic tumor evolution in distinct spatial territories of the breakout lesion, we performed WGS of MM cells from 6 selected regions as well as from the paired rBM of patient P02 (Fig. 5D-E and Fig. S9C). This analysis revealed gain(1q21) and *KRAS*^*G12A*^ as driver events for the common ancestor at the site of the breakout lesion, which was present at the minor subclonal level in the matched rBM stamp. Within the breakout lesion we found two major evolutionary subclonal branches (Fig. 5D-E and Fig. S9C). Branch 1, which included a *HOXD3*^*E333D*^ mutation, was dominant in region R1, present at the minor subclonal level in adjacent regions R2 and R5, and barely detectable in distally located regions R10, R14, and R16 (Fig. 5D). In contrast, branch 2, which was defined by the driver event *TP53*^*T284fs*^ (Fig. 5D), dominated the distally located regions R10, R14, and R16, indicating ongoing diversification and subclones under positive selection in the breakout lesion (29). Notably, the observed spatial genomic divergence coincided with the spatial heterogeneity of T cell clones (Fig. 5B, 5D-E). For instance, TCR clone #3 was exclusively present in regions enriched for tumor subclones from Branch 1, while TCR clone #2 and #14 were enriched in regions mostly containing tumor subclones from Branch 2 (Fig. 5B, 5D-E). However, some differentially enriched TCRs were also observed between regions without detectable heterogeneous mutations, e.g. regions 14 and 16, suggesting that a combination of genomic and microenvironmental factors may underlie TCR heterogeneity.

To further investigate a potential relationship between genomic diversification in tumor cells and clonal T cell expansions, we performed spatially resolved bulk WGS and TCR sequencing of 1-6 regions of breakout lesions and paired rBM stamps in 8 additional patients, corresponding to the same patients included in the extended flow cytometry analysis (Fig. 5F-I and Fig. S10). These analyses confirmed extensive intra-lesion heterogeneity at the genomic and TCR level, with enriched expanded T cell clones in breakout lesions compared to paired rBM stamps in 6 out of 8 patients (Fig. S10E).

In line with the single-cell sequencing data (Fig. 3), there was a trend towards reduced TCR diversity in BoLs, which correlated with the fraction of CD39+/PD1+ CD8 T cells (Fig. S10F-G). Notably, there was also a correlation between the number of enriched TCR clones (FC≥10) and the degree of spatial subclonal tumor heterogeneity in paired comparisons between breakout lesions and rBM stamps or between regions from the same lesion, supporting an association between genomic myeloma evolution and adaptive T cell responses (Fig. S10H, see Methods).

These findings collectively reveal that breakout lesions are critical hubs for concurrent genomic tumor evolution and spatial diversification, likely playing a significant role in expanding the heterogeneity of anti-tumor immune responses.

### Architectural principles of breakout lesions

**Fig 6:**
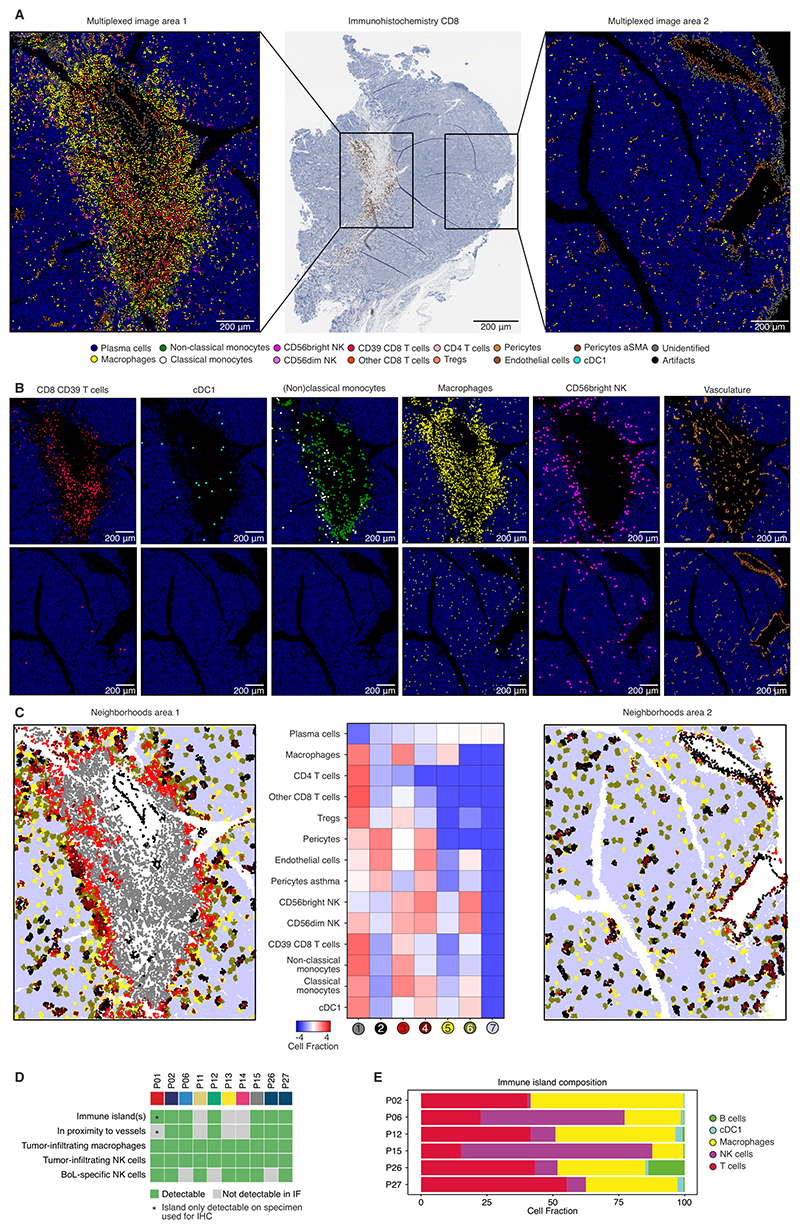
Immune islands as sites of tumor-immune interactions in breakout lesions. A) Multiplexed imaging of the breakout lesion of patient P02. An immune island (area 1, left panel) and a representative region of the surrounding plasma cell-dominated areas (area 2, right panel) are shown. Cells are colored according to their cell type. In the middle panel, a consecutive slide was stained for CD8 T cells using immunohistochemistry. B) Location of selected cell types in the immune island (upper panel) and the plasma cell-dominated area (lower panel) with color code from (A). Except for the cell type of interest and plasma cells (blue) all other cell types are masked. The dot size of the highlighted cells has been increased to improve visibility. C) Neighborhood analysis based on windows of 10 cells. The neighborhoods are shown for area 1 (left) and area 2 (right). Middle: Heatmap depicting the enrichment score for cell types within each neighborhood. The color code for the neighborhoods is shown below the heatmap. D) Summary of key features observed in breakout lesions of 10 patients using multiplexed imaging data. E) Cellular composition of the analyzed immune islands per patient.

Our spatially resolved analysis revealed an unbalanced distribution of immune cells across distinct subregions of breakout lesions. To further explore this phenomenon, we first performed immunohistochemistry (IHC) on 21 breakout lesions, and their paired rBM stamps to identify CD8 T cells (CD8), monocytes/macrophages (CD68), and plasma cells (MUM1). Notably, 9 out of 21 breakout lesions, including those from the two patients with expanded antigen-experienced T cell clones, displayed nodal immune infiltrates surrounded by large territories dominated by plasma cells, which we termed “immune islands” and “plasma cell-dominated areas”, respectively (Fig. 6A and Fig. S11A). Notably, we did not observe a comparable pattern in paired rBM stamps (Fig. S11B), in line with the distinct makeup of the immune ecosystem of breakout lesions as described throughout the manuscript.

To map the spatial architecture of immune islands and plasma cell-dominated areas in more detail, we performed highly multiplexed immunofluorescence imaging of a breakout lesion from patient P02, which included a large immune island and pronounced expansion of site-enriched T cell clones (Fig. 6). For this purpose, we designed a panel to spatially map all major cell types resident in the breakout lesion, including plasma cells, macrophages, CD56bright NK cells, and CD39+ CD8 T cells (Table S5). In total, we identified 14 distinct cell types (Fig. 6A-B, Fig. S11C). To analyze interactions among the cell types, we calculated cellular neighborhoods (CNs), defined as regions with recurrent combinations of cell types (Fig. 6C) (30). For this purpose, we analyzed windows containing the 10 closest spatial neighbors of each cell and clustered these windows according to their cellular composition (30). In total, we defined 7 CNs recapitulating different components of the immune island and the plasma cell-dominated areas. These included the center of the immune islands (CN-1), the rim of the islands (CN-3), endothelial neighborhoods (CN-2, CN-4), the plasma cell-dominated areas (CN-7), as well as two neighborhoods within the plasma cell-dominated areas, which showed an enrichment for macrophages (CN-5), and NK cells (CN-6), respectively.

Consistent with our previous spatial analyses and IHC, CD4 and CD8 T cell subsets were almost exclusively present in the center of the immune islands (CN-1), were depleted at island rims (CN-3), and rarely infiltrated the plasma cell-dominated areas (CN-7) (Fig. 6A-C). The majority of CD8 T cells exhibited an antigen-experienced phenotype, characterized by the co-expression of CD39, LAG3, and TIM3, and were actively expanding, as indicated by Ki67 positivity (Fig. S11D), in line with our flow cytometry and CITE-seq data. cDC1s were observed in close proximity to CD39+ CD8 T cells, suggesting ongoing antigen presentation, further supporting a model in which immune islands in breakout lesions act as active sites of tumor-immune interactions. Notably, a large blood vessel was located at the center of the immune island, likely serving as an entry point for immune cells from the circulation. Moreover, vascular structures were frequently observed at the border between the immune island and the plasma cell-dominated areas, suggesting neovascularization as a consequence of tumor-immune interactions, in line with our single-cell data.

Myeloid cells displayed various phenotypes highly dependent on their spatial localization. Consistent with our previous analysis, non-classical monocytes were highly enriched in the immune island and localized primarily to the island rim in close proximity to, but not infiltrating the plasma cell-dominated area (Fig. 6B). Classical monocytes were less abundant, displayed a similar spatial pattern, but could also be observed in or close to smaller blood vessels. In contrast, M2-like tissue-resident macrophages (CD206+) were observed in the immune islands as well, but also homogeneously infiltrated the plasma cell-dominated area (Fig. 6B, Fig. S11C). Similarly, NK cells, especially CD56bright NK cells with high GZMB expression, migrated from the immune islands and infiltrated the plasma cell-dominated area homogeneously, in line with their lesion-specific expansion and phenotypes (Fig. 6B, Fig. S11C).

We then expanded our multiplex imaging to include 9 additional breakout lesions with sufficient residual material. This analysis confirmed several key features of breakout lesions identified in patient P02, but also revealed substantial interpatient heterogeneity (Fig. 6D). Multiplex imaging confirmed the presence of focal accumulations of immune cells detected by IHC, except in one patient (P01), where different specimens from the same lesion were used for IHC and multiplex imaging due to limited material (Fig. 6D-E). Although the size, composition, and morphology of the immune islands varied (Fig. 6E), they consistently showed an enrichment of CD8+ T cells compared to the surrounding tumor-dense areas and were located in close proximity to blood vessels (Fig. 6D and Fig. S12A). Solitary tumor-infiltrating macrophages and NK cells were observed in all 9 samples and the breakout lesion-specific CD56bright NK cell phenotype (CD7+/CD56++/GZMB+/CD16-) was detected in 6 of them (Fig. 6D and Fig. S12B-C). These analyses demonstrate that although there is considerable heterogeneity between breakout lesions, several key features appear to be conserved in most of them.

Together, these data reveal the distinct spatial organization of MM breakout lesions, which differ markedly from their bone marrow-confined counterparts, featuring immune cell-rich islands and plasma cell-dominated regions as major compartments. Immune islands are key sites of T cell expansion and neovascularization, while specific NK cell and macrophage populations are capable of infiltrating the plasma cell-dominated areas.

## Discussion

The BM microenvironment plays a pivotal role in the development, progression and treatment of MM (31). However, most studies focus on samples collected from a randomly selected single site of the pelvis, regardless of regional disease evolution (6, 32). Here, we have systematically compared such pelvic samples with BM-confined (intramedullary) lesions and so-called breakout (paramedullary) lesions that disrupt the cortical bone and grow as soft tissue masses. Jointly, these analyses uncover the disruption of the cortical bone as a key event in the pathogenesis and tumor immunity of MM. Significantly, the immune and stromal compartments within breakout lesions exhibit a series of major adaptations not previously reported to this extent in MM. Interestingly, several of these phenocopy those previously recognized in solid tumors. This encompasses macrophages with hallmark traits of TAMs, such as *TREM2* and *FOLR2* expression (25, 33–35), the development of T and NK cells with an antigen-experienced phenotype (23, 36, 37) and T cells with a phenotype similar to recently described tumor specific T cell signature in melanoma (26). As these adaptations were not observed in paired BM counterparts, they are likely consequences of sustained tumor-immune interactions and changes from a medullary to a non-medullary immune microenvironment.

Changes in the BM immune TME have recently also been reported in advanced stages of plasma cell dyscrasias, including an increase in NK cells, Tregs, and immunosuppressive macrophages, as well as a depletion of memory T cells in active MM compared to precursor conditions, albeit to lesser extent (31, 38, 39). Notably, patients with early relapse show an increase in M2 macrophages and CD8 T cells with an exhaustion signature (40), and higher levels of such CD8 T cells have recently been associated with poor response to immunotherapy (41, 42), suggesting that changes within the TME of breakout lesions are consistent with high-risk and/or treatment-resistant disease. This interpretation is supported by the recent observation that the presence of multiple breakout lesions is an independent prognostic factor in MM (14). Correlating immune parameters of breakout lesions with clinical outcome will be an important next step, but the difficulty in obtaining such samples, the limited clinical follow-up when new sample processing procedures are applied, and the presence of potential confounders such as subclonal heterogeneity within breakout lesions make such an analysis challenging.

Spatial analysis revealed that the organizational principles of breakout lesions differ drastically from BM sites with a diffuse infiltration pattern, with immune islands and plasma cell-dominated areas often dominating the breakout lesion landscape. Immune islands are sites of immune cell expansion and interaction with malignant plasma cells surrounding the immune islands in tumor cell-dominated areas. While immune islands resemble to some extent tertiary lymphoid organs (TLOs) observed in solid tumors (43), they are devoid of B cells and dominated by T, NK and myeloid cells. Interestingly, immune islands typically formed around blood vessels and contained cDC1s, in line with recent findings that these cells promote the infiltration of antigen-specific T cells in MM and other tumors (44, 45). In contrast to T cells, monocytes and dendritic cells, which were mainly observed within the immune islands, distinct populations of NK cells and macrophages were capable of homogeneously infiltrating the plasma cell-dominated areas. However, their exact role in myeloma pathogenesis and immune regulation remains to be clarified.

Our data suggest that breakout lesions may be a hotspot for subclonal evolution that likely drives diversification of the T cell repertoire. Therefore, breakout lesions may play a key role in both the generation of tumor heterogeneity and T cell immune responses. However, in addition to genomic events, micro- and macroenvironmental factors may also contribute to the differential immune cell responses in breakout lesions. Putative tumor-specific T cells with signatures of exhaustion and antigen experience, including high expression of immune checkpoint molecules, were locally restricted, which is in contrast to a systemic anti-tumor immunity, recently described in breast cancer (37). This may explain why checkpoint inhibition is a promising therapeutic strategy in several solid tumors (46, 47), but has shown rather disappointing results in MM (48, 49). Yet, functional imaging is required to monitor localized therapy responses (50), which are possible given the strong differences in tumor immunology between breakout lesions and diffusely infiltrated BM. Furthermore, while less favorable outcomes after T cell re-directing therapies, including bispecific antibodies and CAR-T cell therapies, are mainly associated with EMD (51–54), our results suggest that the distinct genomic and TME features of paramedullary disease (i.e. breakout lesions) need to be considered when linking EMD biology to clinical outcomes in future studies to unravel the mechanisms underlying poor survival.

Jointly, our study highlights the importance of studying the co-evolution of myeloma and its TME within and outside the BM. We uncover breakout lesions as major sites for tumor evolution and immune cell diversification, representing a key event in myeloma pathogenesis and tumor immunity with potential therapeutic implications.

## Materials and Methods

### Study design

The aim of this study was to elucidate the early processes associated with BM independence in MM by characterizing the tumor-immune interactions in breakout lesions compared to rBM samples. For this, we used a multi-omics approach.

### Patients

We included 54 samples from 16 NDMM patients fulfilling the International Myeloma Working Group (IMWG) criteria for treatment (4) (Fig. S1A-B). For validation purposes, we used trephine biopsies and/or WGS data from 21 additional patients, including 19 patients from a recently published study (11) (Fig. S1B). Patients’ characteristics, follow-up data, the origin of samples and the analyses, which were performed with the samples, are shown in Data files S1-S2 and Fig. S1B. Informed consent for sample collection and processing in accordance with the Declaration of Helsinki was obtained for all cases included in this study that had been approved by the Heidelberg University Medical Faculty ethics review board (S278-13).

### Medical imaging

Osteolytic lesions were diagnosed using whole body CT. CT-guided sampling was performed using a Siemens Emotion 16 CT (Siemens Co., Erlangen, Germany). Surgical resections were performed in patients with clinical indication for stabilization of the spine.

### Sample preparation

Focal lesions for CT-guided biopsies were chosen according to accessibility and minimal risk for the patient. In case of multiple eligible lesions, the best accessible lesion was selected. CT-guided biopsies were performed in a separate session within one week after collection of the rBM from the iliac crest. Mononuclear cells (MC) from rBM aspirates and PB were isolated using the Ficoll–Paque method. BM MM cells were enriched using immuno-magnetic CD138-positive selection (Robosep, Stemcell Technologies). The CD138-positive and -negative fraction were either stored in Qiagen RLT buffer (bulk sequencing) at -80°C or viably frozen in 10% dimethylsulfoxide (sc sequencing and flow cytometry) at -150°C. PBMC dry pellets were stored at -20 °C. Samples from surgical resections and stamps were minced and enzymatically digested for 15 min at 37°C in MEM alpha Medium (Gibco, USA) containing 1 mg/ml Collagenase II, 0.8mg/ml Dispase (both Gibco, USA) and 0.1mg/ml DNAse I (SigmaAldrich, USA). Cells were released by pipetting repetitively (30x). The reaction was stopped using a quenching buffer (Calcium-free PBS + 5mM EDTA + 2% FCS). To enrich MM cells, the cells were sorted (CD38-high, HLA-DR-negative, CD3-negative, CD45-positive) using a FACSAria (BD Biosciences). Dead cells were identified using eFluor-506 (ThermoFisherScientific, USA). Sorted cells were stored in RLT buffer (Qiagen, Hilden, Germany) at -80 °C. The same gating strategy as for CITE-seq was used (Fig. S5A). Sample availability and processing methods are shown in Data file S2.

### Flow cytometry

Rainbow Calibration Particles (BD Biosciences) were used for voltage adjustment. Compensation controls were prepared using UltraComp eBeads™ Compensation Beads (Thermo Fisher Scientific). Efluor compensation controls were prepared using BMMCs or PBMCs. Antibody master mixes were prepared in Brilliant Stain Buffer Plus (BD Biosciences). Antibody panels are shown in Tables S1, S2, and S4. Cryopreserved samples were thawed at 37ºC, resuspended in RPMI with 10% FCS, centrifuged at 500 g for 5 min and the pellet resuspended in 400 µL FACS buffer (1x DPBS, 5% FCS, 0.5 mM EDTA). All following steps were performed in a Nunc™ 96-Well Polystyrene Conical Bottom MicroWell™ Plate. First, cells were centrifuged at 500 g for 5 min, and the pellet resuspended in a total volume of 30 µL with eFluor-506 fixable viability dye (eBioscience, 1:1000 dilution) and FcR Blocking Reagent (Miltenyi Biotec, 1:20 dilution). After 5 min at RT in the dark, 50 µL antibody mastermix was added. After 15 min at 4ºC in the dark, 120 µL FACS buffer were added, the plate centrifuged at 500 g for 5 min, and the pellet resuspended in 200 µL FACS buffer. A third washing step with 200 µL FACS buffer was performed, before cells were resuspended in 30 µL FACS buffer and transferred to a 1.2 mL Individual Reaction Tube (Starlab) for flow cytometry using a BD FACSymphony™ A5 Cell Analyzer. A reference control sample was measured per plate.

### Single-cell RNA-sequencing including CITE- and VDJ-sequencing

Cryopreserved samples were thawed at 37ºC, resuspended in 10 mL RPMI with 10% FCS, and centrifuged at 300 g for 5 min. The pellet was resuspended in 10 mL RPMI with 10% FCS. Cells were centrifuged at 300 g for 5 min, and the pellet resuspended in 50-100 µL PBS plus Calcein (0.1 µM 1:100 from 10µM diluted stock) and efluor-506 fixable viability dye (eBioscience, 1:1000). After 5 min at RT, 100-200 µL antibody master mix for FACS sorting (Table S3) were added. Individual samples were hashed by adding 0.1 µg TotalSeq of the respective anti-human Hashtag 1-4 and 6-9 (Biolegend). After 15 min at 4ºC, 5 mL FACS buffer (1 x DPBS, 5% FCS, 0.5 mM EDTA) was added and the tube centrifuged at 300 g for 5 min, and the pellet resuspended in 300 µL FACS buffer. Sorting was performed with a BD FACS Aria. Gating strategy is shown in Fig. S5A. For each cell population and sample, a maximum of 10,000 events (range=3500-10000 per population, CD90+CD34+ all cells) were sorted into 1.5 mL Protein low binding Eppendorf tubes prefilled with 150 µL Cell Staining Buffer (Biolegend). Sorted cells were pooled into batches, where paired samples from the same patient were in different batches. Each batch contained approx. 130,000 sorted cells, which were centrifuged at 300 g for 5 min and the pellet resuspended in 25 µL Cell Staining Buffer. Subsequently, 2.5 µL of Human TruStain FcX Blocking reagent (Biolegend) was added to the pooled cell suspension and incubated for 10 min at 4°C. After adding 25 µL of reconstituted TotalSeq™-C antibody cocktail (Biolegend), cells were incubated for 30 min at 4°C. Cells were washed 3x with 3.5 mL Cell Staining Buffer and resuspended in PBS and loaded onto three 10x GEMs (22,000-24,000 cells per GEM) per batch. Immunoprofiling was performed according to the Chromium Next GEM Single Cell 5′ Reagent Kit v2 (Dual Index) user guide with feature barcode technology for cell surface protein & immune receptor mapping (10x Genomics; CG000330 Rev A). Generated gene expression (GEX) and protein (ADT) libraries were paired-end sequenced on the NovaSeq 6000 S4. Generated V(D)J libraries were paired-end sequenced on the NextSeq 550.

### Whole genome sequencing and variant calling

DNA was isolated using the Allprep Kit (Qiagen). WGS libraries were prepared with the Illumina TruSeq Nano DNA kit and sequenced on a NovaSeq 6000 S4 flowcell (paired-end 150 bp) to an average coverage of 85x for tumor and 43x for germline samples. Raw data was processed and aligned to human reference genome build 37 version hs37d5 using the DKFZ OTP WGS pipeline (55). Copy number aberrations (CNAs) were identified using ACEseq (v1.2.8-4) (56), indels using platyphus (v2.4.1-1) (57) and single nucleotide variants (SNVs) using samtools mpileup (v1.2.166-3) (58). For SNVs additional filtering was applied, including blacklist filtering (57), fpfilter (https://github.com/genome/fpfilter-tool) and removal of SNVs in immunoglobulin regions. For SNVs, which were only called in one of the paired samples, Rsamtools (v2.6.0) was used to determine the number of variant reads in both samples. Manual somatic variant refinement was performed using IGV (v2.7.2) (59) according to published recommendations (60). The cancer clonal fraction (CCF) was calculated as described (61). To construct phylogenetic trees, we first distinguished positive subclonal selection from neutral tails using the R package mobster v1.0.0 (29). However, SNVs classified as tail mutations but assigned to cluster C1 in a paired sample were not removed. We then used mobster to infer subclonal architectures and visualize phylogenetic trees. For annotation of branches, we considered recently identified driver genes (62, 63). Indels and CNAs were assigned to branches manually.

### Preprocessing and analysis of flow cytometry data

Live single cells were gated and flow cytometry data was compensated using FlowJo (v.10.8.4) and subsequently analyzed using the Spectre (v.1.0.0) and Seurat (v.4.9.9.9050) R packages. In brief, compensated scaled values exported from FlowJo were transformed using an inverse hyperbolic asinh transformation with a cofactor = 500. For batch correction, the CytoNorm model (v.0.0.10) was trained using 600 cells per sample, including reference controls, and used to normalize each marker value. Cells were then clustered using FlowSOM (v2.4.0) and automatically annotated in a cluster-free manner using a linear discriminant analysis model (LDA) via MASS (v7.3-59) R package, trained with a manually annotated subset of the dataset. Dimensional reduction and visualization in a UMAP space was performed using the sketching approach from Seurat. Representative cells per day of acquisition were sketched (100,000 cells/day for the identity panel and 50.000 cells/day for the T cell panel) and subsequently integrated using the reciprocal PCA (RPCA) approach. Cell clustering was performed in the sketched subset prior to projection of the whole dataset into the integrated space. Cell clusters representing doublets or artifacts were removed. Cell annotations were manually corrected for ambiguous clusters based on known marker gene expressions. CD4 was not considered for clustering and dimensionality reduction as it was affected by enzymatic digestion. Only one of multiple regions was initially included in the analysis. Additional regions were preprocessed as described above.

### Preprocessing and analysis of CITE-seq data

CellRanger multi (v7.0.0) was used for the alignment of demultiplexed libraries to reference genome GRCh38 (refdata-gex-GRCh38-2020-A) and the generation of the feature barcode matrices. For the TCR libraries, reference refdata-cellranger-vdj-GRCh38-alts-ensembl-7.0.0 was used. The count matrices were loaded into R (v4.2.2) by using the standard Seurat (v5.0.1) parameters. Immunoglobulin and TCR genes were removed from the gene expression matrix and added as additional assays in the Seurat objects. Antibodies of isotype controls (Mouse IgG2b kappa, Rat IgG2b kappa, Mouse IgG2a kappa, Mouse IgG1 kappa isotype) were removed from the ADT assay. Demultiplexing was performed based on the Hashing antibodies with HTODemux and based on SNPs using vireo (64, 65). scDblFinder (66) was used to call cell doublets. Cells with >5% mitochondrial RNA and <400 expressed genes were removed (Fig. S5B). Samples were annotated for patient as well as sample location and then merged together. GEX Normalization was done using SCtransform (67) and confounders such as mitochondrial counts or cell cycle stages were regressed out using the “vars.to.regress” argument. Protein levels were normalized with CLR. All samples were clustered together based on Seurat k-nearest neighbors clustering based on the SCT normalized gene expression values with a resolution of 0.5 and cells were embedded into a two-dimensional space using Uniform Manifold Approximation and Projection (UMAP) (68) (Fig. S5C). The dataset was split into tumor and TME using typical plasma cell markers (Fig. S5D). The cell type assignment of the TME was done manually based on the gene (GEX) and protein (ADT) expression of typical cell type markers (Fig. S6). For a more detailed cell type annotation, a subset of each compartment was created, SCT normalized, clustered and embedded in a separate UMAP space. CD4 and CD8 T cells were differentiated using scGate (64) with gene expression levels of both markers. As CD4 protein expression was affected by enzymatic digestion, CD4 gene expression was used to detect CD4 T cells. Differential expression analysis was performed with the standard Seurat function Find(All)Markers (parameters: min.pct = 0.25, logfc.threshold = 0). Gene expression signatures were calculated with the Seurat function AddModule Score (https://satijalab.org/seurat/index.html) by using recently published transcriptional signatures to determine the level of T cell exhaustion (28), T cell neoantigen-reactivity (27), NK-cell exhaustion (23), NK-cell cytotoxicity (24), extracellular matrix production (Gene ontology geneset) and M2-like macrophages (25, 69). VDJ filtered_contig_annotations files were loaded into R, combined from all channels and mapped to the Seurat object by using the scRepertoire package (v2.0.0) (70). T cell clones, which made up ≥1% of the total T cell population and showed ≥10 cells in one of the paired samples, were called expanded. T cells with incomplete receptor sequences were merged with the corresponding T cells with complete sequences. Known epitopes of expanded T cell clones were retrieved from the curated databases VDJdb (71) and McPas-TCR (72).

#### Deep/Bulk TCR-sequencing

DNA was isolated using the Allprep Kit (Qiagen) and CDR3β and TRBV sequencing was performed by immunoSEQ® (Adaptive Biotechnology, Seattle WA). Data were analyzed using the immunoSEQ ANALYZER 3.0 (Adaptive Biotechnology, Seattle WA), with only productive CDR3β rearrangements used for TCR frequency calculations. TCR clones representing ≥1% of the total T cell population and showing a fold-change ≥10 between breakout lesion and rBM stamp or between regions within the same lesion were considered enriched.

### Immunohistochemistry

Representative tissue blocks containing MUM1-positive myeloma cells were selected. The blocks were sectioned with a standard microtome at 2 µm thickness. Subsequently, the slides were dried overnight at room temperature. Immunohistochemical staining for MUM1 (mouse anti-human clone Mum1p, Agilent DAKO, Santa Clara, USA), CD68 (mouse anti-human clone KP-1, Roche Ventana, USA), and CD8 (rabbit anti-Human, clone SP57 Roche Ventana, USA) was performed on consecutive tissue sections using the automated immunostainer Ventana Benchmark Ultra (Roche, USA). For 8 patients, CD68 (DAB) and CD8 (FastRed) were co-stained on the same section. Images of stained slides were acquired at RT using the Aperio AT2 slide scanner at 40x magnification (Leica 20x/0.75NA Plan Apo objective with 2x automatic optical magnification changer; resolution 0.25 μm/pixel) and the manufacturer’s acquisition software suite (Leica Biosystems, Nussloch, Germany, version 102.0.7.5). Appropriate focus points were set using the automatic focusing strategy of the acquisition software. Focus points were corrected when set in areas without tissue. Images were analyzed using the QuPath software (v0.3.2). Therefore, the images were imported using the Bioformats builder. A final check of all stained and detected cells was performed by an expert pathologist.

### Multiplex imaging

FFPE tissue sections (2 µm) were incubated at 60°C for 30 min and deparaffinized in Histo-Clear II for 20 min before being rehydrated in a graded alcohol series (ethanol:deionized water, 100:0, 100:0, 90:10, 70:30, 50:50, 70:30; 3 min each). The slides were washed twice in 20 mM Tris, 150 mM NaCl, pH 7.6 (TBS) for 5 min and antigen retrieval was performed in Tris-EDTA buffer (pH 9) with 0.05% Tween-20 for 20 min at 100°C. Following cooling to RT, the slides were washed twice in TBS for 5 min. Autofluorescence was quenched by submersing the slides in PBS with 2% H_2_O_2_ and 20 mM NaOH for 30 min at RT and concomitant light exposure using a 10,000 lux LED lamp (RHM). Following two washes in TBS for 5 min, the tissue was blocked with 3% bovine serum albumin (BSA) and 2.5% goat serum in TBS for 30 min at RT. The samples were stained with anti-MUM1 (clone Mum1p, mouse) at 1:50 and anti-FOXP3 (clone SP97, rabbit) at 1:50 in 1% BSA in TBS overnight at 4°C and washed twice with TBS for 5 min. Multiplex immunofluorescence was performed using the antibody panel described in Table S5 and the Lunaphore COMET™ platform (Biotechne). For the validation experiment, PAX5 was replaced with CD20 and CD7 was added to the panel (Table S5). The COMET™ platform is equipped with an LED-based widefield microscope and 20X objective that acquires images in three fluorescent channels (DAPI, TRITC, Cy5), providing subcellular resolution and an image pixel size of 0.23 µm (73).

Image registration and background subtraction were performed in the Lunaphore Viewer (v1.0.3). Single cells were segmented and quantified using the MCMICRO pipeline (74), module mesmer (75) and MCQUANT. Cell segmentation was based on the nuclear channel and typical membrane markers for each cellular compartment. For analysis of the breakout lesion from patient P02, two representative regions (1545 x 1631 µm) were selected. Marker expression levels were z-normalized and an initial round of Leiden-based clustering (n_neighbors = 10, resolution = 1.5) was performed with all markers except for PAX5, Ki67, PD1, CD39, TIM3 and LAG3 using the scanpy Python package (76, 77). Each cluster was assessed for its cell type composition on the basis of marker expression and overlays of the cells in each cluster onto image hyperstacks using CODEX scripts for ImageJ/Fiji (https://github.com/bmyury/CODEX-fiji-scripts). Clusters expressing CD38, CD138, and MUM1 were labeled as plasma cells. All remaining cells were re-clustered. Clusters were merged, split and/or further subclustered until all possible immune cell subsets were identified (Fig. S11C). Neighborhood analysis was performed as described (30, 78). In brief, the 10 nearest neighbors of each cell were determined on the basis of their Euclidean distance of the x and y coordinates resulting in a window per cell. These windows were grouped using k-means clustering based on the cell type proportions within each window. Each cell was given the neighborhood label defined by its surrounding ‘window’. k = 14 was selected on the basis of overlays of the neighborhood assignments with the original images. Related neighborhoods were merged, resulting in 7 distinct neighborhoods. For the validation set, data analysis was performed as described above, with the addition of the scimap Python package (79). To determine the composition of immune islands, rare cell types (e.g. cDC1 or B cells) were counted manually.

### Statistical analysis

Statistical analyses were carried out using the R software package v4.3.3 and v4.4.0. Group comparisons of continuous variables were done using the two-sided Wilcoxon rank sum test for unpaired samples and the two-sided Wilcoxon signed rank test for paired samples. P-values were corrected for multiple comparisons using the Benjamini-Hochberg method. Correlation coefficients were determined using Pearson correlation or linear mixed-effects models. Similarity between correlation matrices was assessed using the Mantel test. Differential abundant cell type frequencies were calculated as a log2(fold-change), adding one cell to each cell type per sample prior to the comparisons to avoid cell frequencies of 0.

## Supplementary Material

Supplementary Materials

## Data Availability

WGS, CITE- and scTCR-sequencing data from this study have been deposited in the European Genome-phenome Archive (EGA) under study identifier EGAS50000000304 and are available on request from the associated Data Access Committee (hipo_daco@dkfz-heidelberg.de) as they contain patient information under controlled access. Access will be provided to commercial and non-commercial parties in accordance with patient consent forms and data transfer agreements. We have an institutional process for handling data transfer requests and aim for a rapid response time. The duration of data access after approval is limited to 36 months. Processed single-cell multi-omics sequencing data will be made available on the Gene Expression Omnibus upon acceptance. WGS data from the validation set (11) are available under EGAS00001006090. Source/raw data for all figures are provided in Data File S7.

## One Sentence Summary

Single-cell and spatial multi-omics analyses identify breakout lesions as a hotspot for tumor-immune interactions and diversification.
