# Supplementary material for: Bone marrow breakout lesions act as key sites for tumor-immune cell diversification in multiple myeloma
Source: Sci Immunol. Author manuscript; Available in PMC 2026 Jun 27. (PMC7619224; doi:10.1126/sciimmunol.adp6667)
Supplement: Supplementary Materials [file EMS214146-suppement-Supplementary_Materials.pdf]

Supplementary Materials

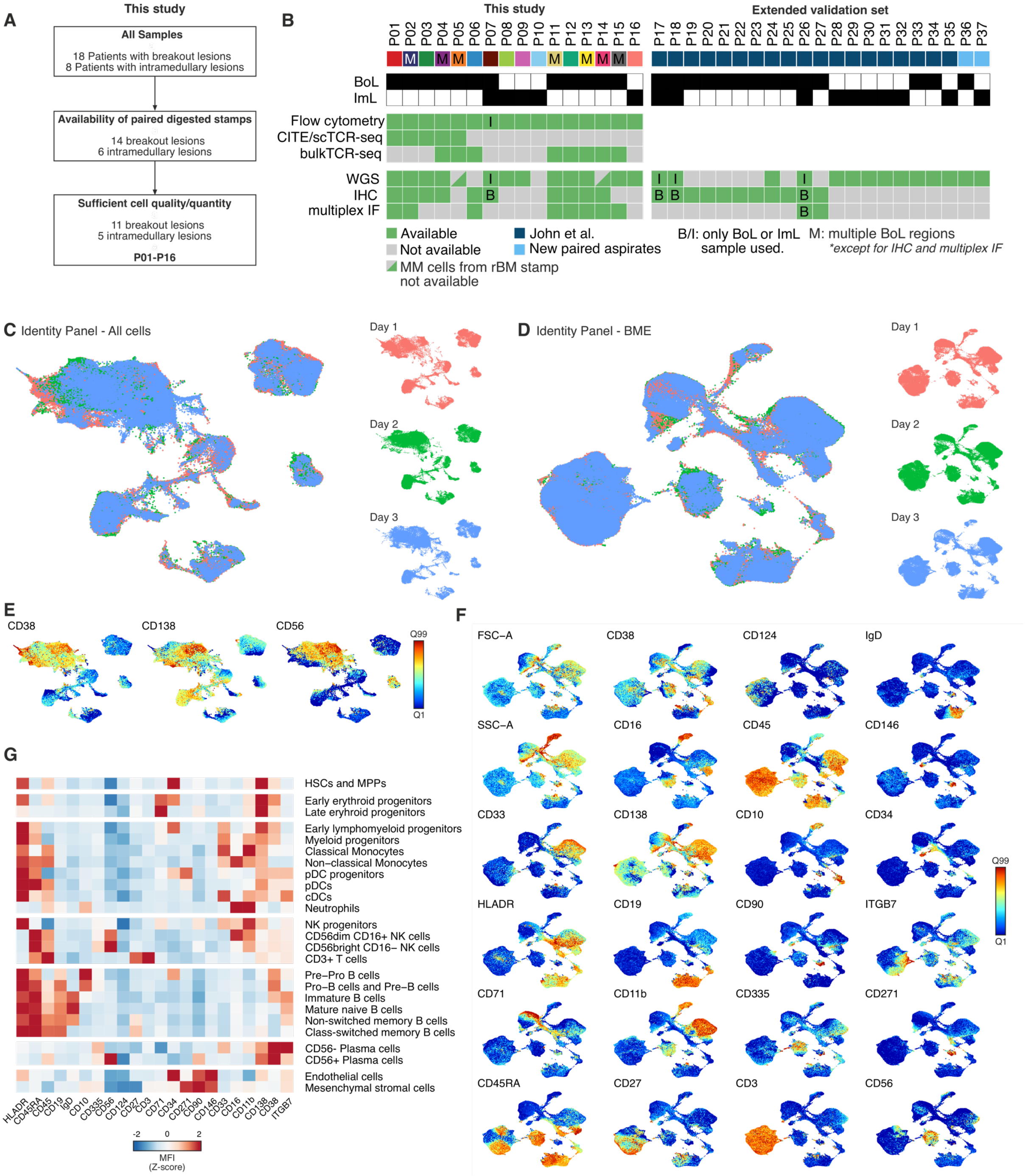

Fig. S1. Study overview and quality control of the "identity" cytometry panel. A) Consort diagram summarizing the selection of samples used in this study. B) Summary of sample availability for the different methodologies used in this study. Sample types per patient used in each method are specified in **Data file S2**. C) UMAP representation of the identity panel (n=150,000 sketched cells) including cells from peripheral blood (PB), random bone marrow (rBM) aspirates and stamps as well as intramedullary lesions (ImL) and breakout lesions (BoL). Cells are coloured per day of acquisition. D) UMAP representation of the identity panel without plasma cells (n=300,000 sketched cells). Cells are coloured per day of acquisition. E) Normalized expression of key plasma cell markers projected onto the UMAP representation of the identity panel. F) Normalized expression of all markers included in the identity panel projected onto the UMAP representation. G) Heatmap displaying the scaled marker expression of the identity panel markers by cell type.

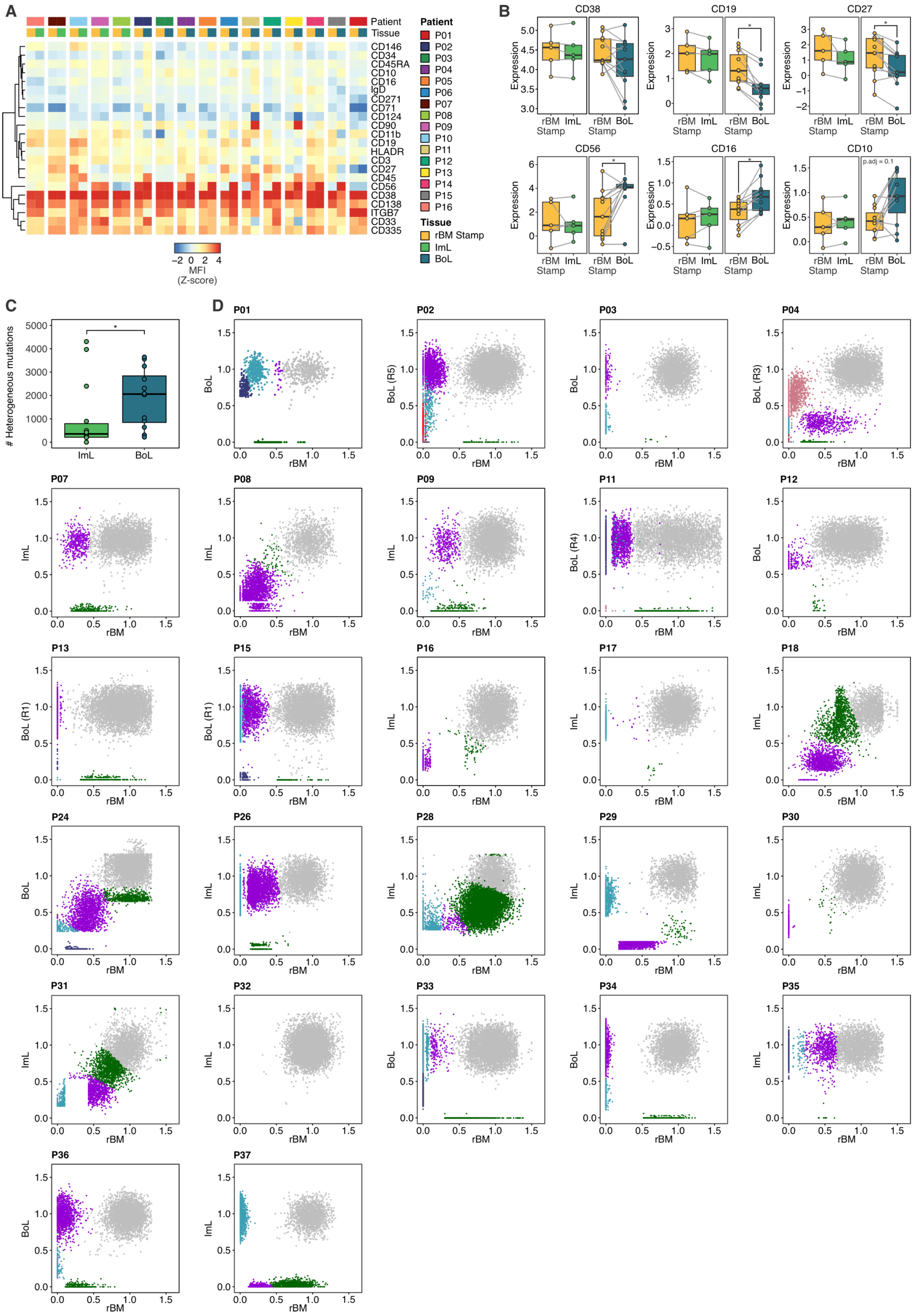
A
Patient
Tissue
CD146
CD34
CD45RA
CD10
CD16
IgD
CD271
CD71
CD124
CD90
CD11b
CD19
HLADR
CD3
CD27
CD45
CD56
CD38
CD138
ITGB7
CD33
CD335
−2 0 2 4
MFI
(Z-score)
Patient
P01
P02
P03
P04
P05
P06
P07
P08
P09
P10
P11
P12
P13
P14
P15
P16
Tissue
rBM Stamp
ImL
BoL
B
CD38
CD19
CD27
CD56
CD16
CD10
Expression
rBM Stamp
ImL
BoL
p.adj = 0.1
C
Heterogeneous mutations
ImL
BoL
D
P01
P02
P03
P04
P07
P08
P09
P11
P12
P13
P15
P16
P17
P18
P24
P26
P28
P29
P30
P31
P32
P33
P34
P35
P36
P37
rBM
BoL
ImL
BoL (R5)
BoL (R3)
BoL (R4)
BoL (R1)

Fig. S2. Plasma cell characteristics and genomic heterogeneity between focal lesions and paired bone marrow. A) Heatmap displaying the scaled marker expression of the identity panel markers in plasma cells from paired random bone marrow (rBM) and focal lesion (either intramedullary (ImL) or breakout lesion (BoL)) per patient. B) Differentially expressed markers in paired rBM and focal lesions (either ImL or BoL). C) Boxplot showing the number of heterogeneous mutations between BoLs and paired rBM compared to ImLs and paired rBM. As heterogeneous mutations, we classified all mutations characterizing subclones that showed more than a 3-fold difference in the median cancer clonal fraction (CCF) between paired samples. We increased the sample size to 14 ImL/rBM and 12 BoL/rBM pairs by including publicly available WGS data of paired aspirates (*11*) (see **Fig. S1B**). D) CCF plots for total (synonymous and nonsynonymous) SNVs for all these patients. For patients with more than one region per lesion, we selected one of them. For patients P05 and P14, no remaining plasma cells from the rBM sample were available for WGS. Mutations are colored according to tumor subclone.

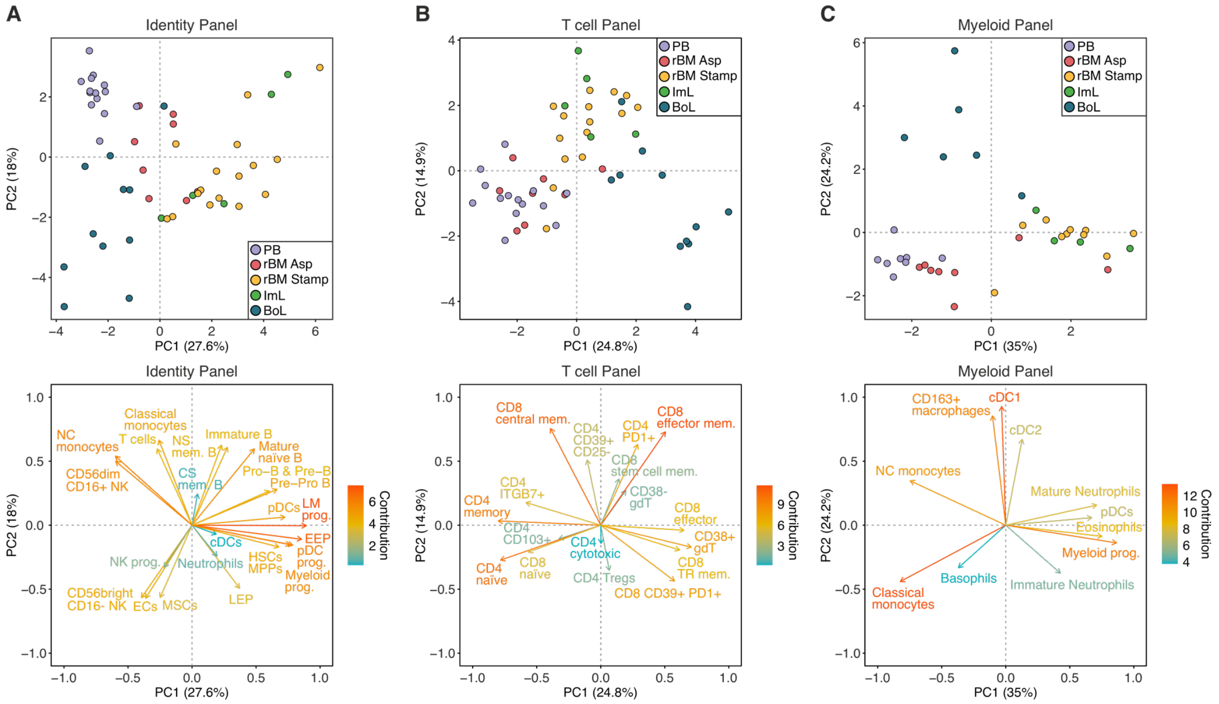

Fig. S3. Principal component analyses of multi-paramentric flow cytometry data. A-C) Upper panels: principal component analysis (PCA) based on cell type fractions obtained from flow cytometry data using the identity panel excluding plasma cells (A), T cell panel (B) and myeloid panel (C). Lower panels: Relative cell type contribution to components PC1 and PC2 to matching plots of upper panels. Arrows indicate directionality of contribution and color and arrow length indicate relative contribution.

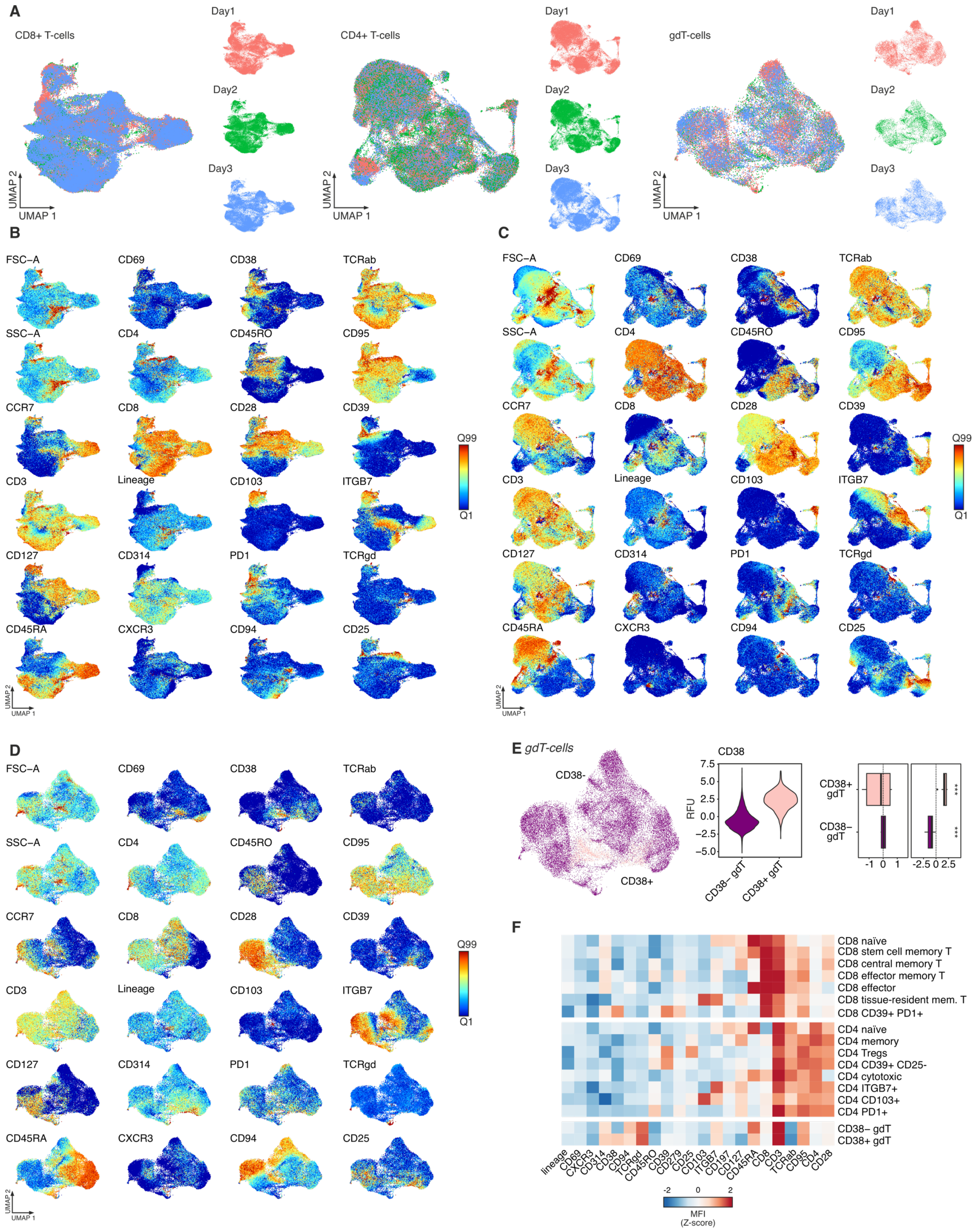
A
CD8+ T-cells
CD4+ T-cells
gdT-cells
Day1
Day2
Day3
UMAP 1
UMAP 2
B
C
D
FSC–A
CD69
CD38
TCRab
SSC–A
CD4
CD45RO
CD95
CCR7
CD8
CD28
CD39
CD3
Lineage
CD103
ITGB7
CD127
CD314
PD1
TCRgd
CD45RA
CXCR3
CD94
CD25
Q99
Q1
E gdT-cells
CD38-
CD38+
CD38
RFU
CD38− gdT
CD38+ gdT
CD38+ gdT
CD38– gdT
F
CD8 naïve
CD8 stem cell memory T
CD8 central memory T
CD8 effector memory T
CD8 effector
CD8 tissue-resident mem. T
CD8 CD39+ PD1+
CD4 naïve
CD4 memory
CD4 Tregs
CD4 CD39+ CD25-
CD4 cytotoxic
CD4 ITGB7+
CD4 CD103+
CD4 PD1+
CD38− gdT
CD38+ gdT
lineage
CD69
CXCR3
CD314
CD38
CD94
TCRgd
CD45RO
CD39
CD279
CD25
CD103
ITGB7
CD197
CD127
CD45RA
CD8
CD3
TCRab
CD95
CD4
CD28
MFI
(Z-score)

Fig. S4. T cell-focused cytometry panel. A) UMAP representation of the CD8 (left, n=138,301 sketched cells), CD4 (middle, n= 150,000 sketched cells) and gamma-delta (gd) (right, n=31,208 cells) T cell subsets including cells from peripheral blood (PB), random bone marrow (rBM) aspirates and stamps as well as intramedullary lesions (ImL) and breakout lesions (BoL). Cells are coloured per day of acquisition. B-D) Normalized expression of all markers included in the T cell panel projected onto the UMAP representation of either CD8 (b), CD4 (C) or gdT (D) T cell subsets. E) Left: UMAP representation of the gdT cells highlighting the subsets identified based on CD38 expression, also depicted as a violin plot (middle). Right: Relative gdT subtypes abundance in ImL and BoL. Data is represented as log2(fold-change) compared to average cell type abundance in paired rBM stamps. F) Heatmap displaying the scaled marker expression of the T cell panel markers by cell type. Statistical analyses in (E) were performed using the Wilcoxon signed rank test, comparing ImL or BoL with paired rBM stamps. **P* <0.05, ***P* <0.01, ****P* <0.001, *****P*<0.0001.

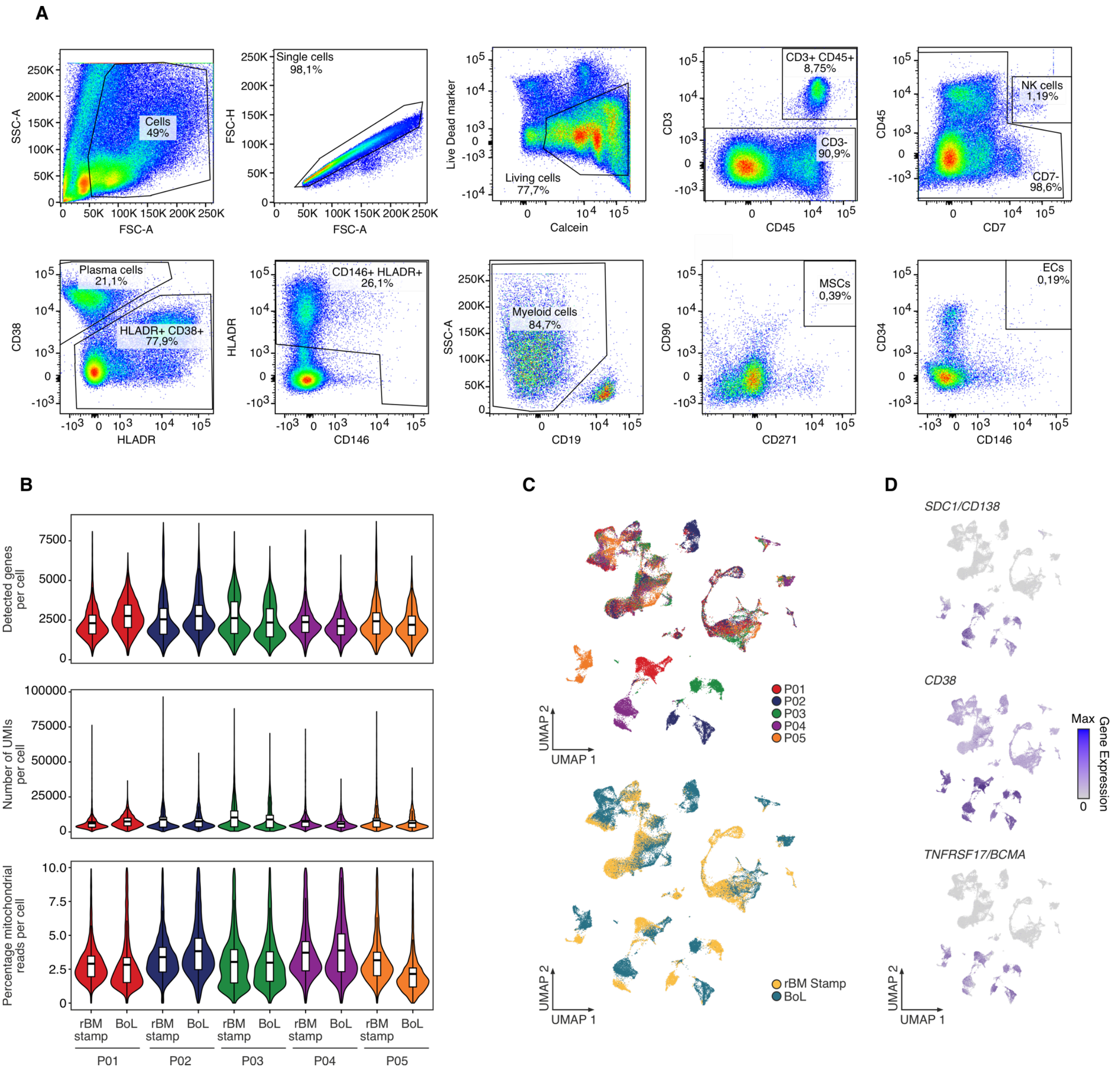

Fig. S5. Sorting scheme, quality control of the CITE-seq data, and genomic heterogeneity. A) FACS sorting scheme of the different cell populations (T, NK, myeloid, plasma and stromal cells) included in the CITE-seq workflow. Cell populations were selected based on the flow cytometry dataset (Fig. 1). T cells were defined as CD3+CD45+, NK cells as CD45+CD7+ (after T cell exclusion), plasma cells as CD38+ HLADR-, myeloid cells as CD146+HLADR+CD19- and stromal/endothelial cells as CD34+CD90+. B) Violin and box-whisker plot of the number of informative genes, number of unique molecular identifier (UMI) transcripts and percentage of mitochondrial reads per single cell per experimental sample to define quality control (QC) thresholds. The boxplots show the median and the interquartile range, while the upper and lower whiskers show the highest and lowest value. C) UMAP representation of the CITE-seq data of 5 patients with paired breakout lesion (BoL) and random bone marrow (rBM). UMAP coloured by patient (top) and location (down). D) UMAP projection of single cells colored by expression of the plasma cell markers *SDC1* (CD138), *CD38* and *TNFRSF17* (BCMA).

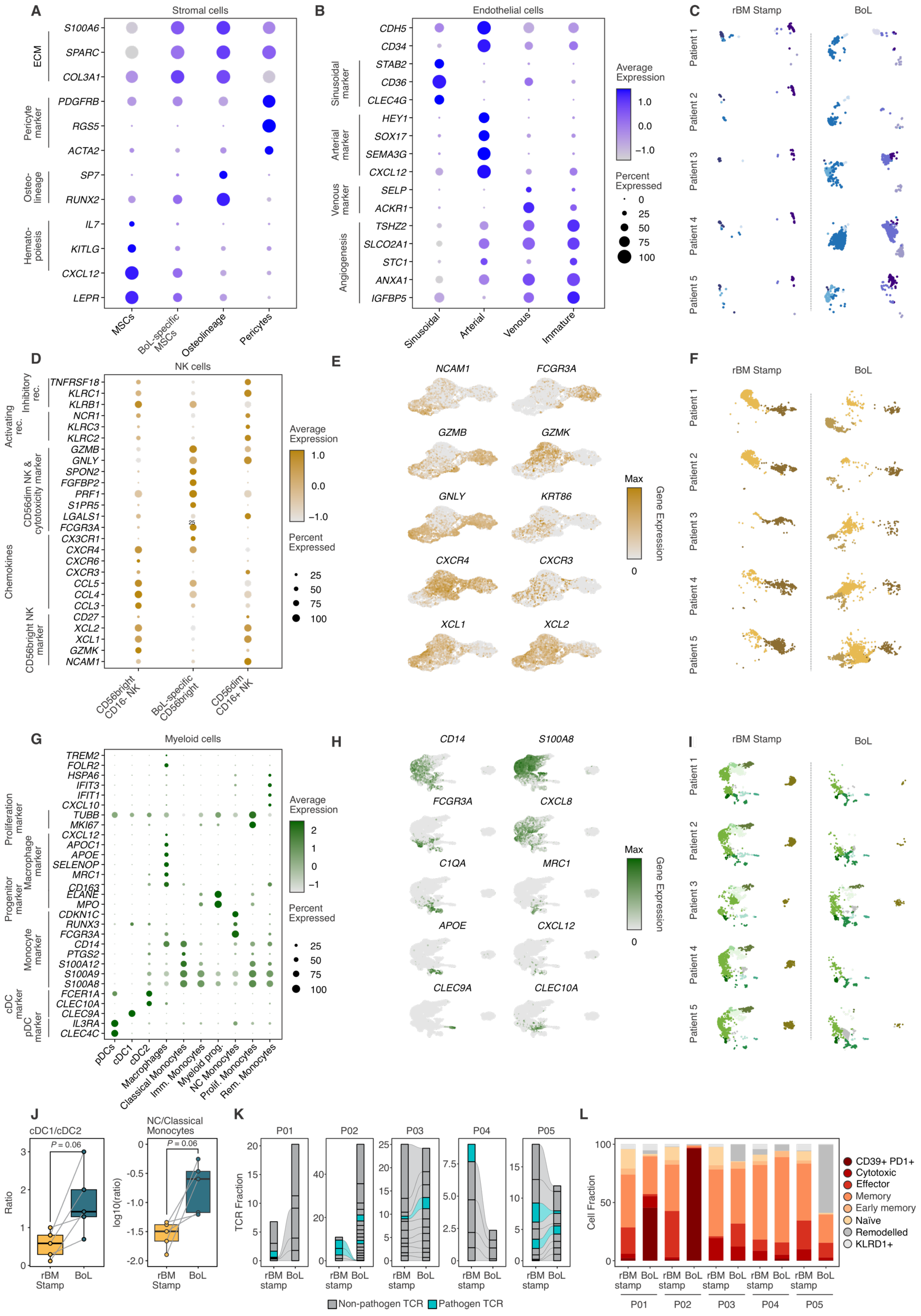
A
Stromal cells
S100A6
SPARC
COL3A1
PDGFRB
RGS5
ACTA2
SP7
RUNX2
IL7
KITLG
CXCL12
LEPR
ECM
Pericyte marker
Osteo-lineage
Hemato-poiesis
MSCs
BoL-specific MSCs
Osteolineage
Pericytes
B
Endothelial cells
CDH5
CD34
STAB2
CD36
CLEC4G
HEY1
SOX17
SEMA3G
CXCL12
SELP
ACKR1
TSHZ2
SLCO2A1
STC1
ANXA1
IGFBP5
Sinusoidal marker
Arterial marker
Venous marker
Angiogenesis
Sinusoidal
Arterial
Venous
Immature
Average Expression
1.0
0.0
−1.0
Percent Expressed
0
25
50
75
100
C
rBM Stamp
BoL
Patient 1
Patient 2
Patient 3
Patient 4
Patient 5
D
NK cells
TNFRSF18
KLRC1
KLRB1
NCR1
KLRC3
KLRC2
GZMB
GNLY
SPON2
FGFBP2
PRF1
S1PR5
LGALS1
FCGR3A
CX3CR1
CXCR4
CXCR6
CXCR3
CCL5
CCL4
CCL3
CD27
XCL2
XCL1
GZMK
NCAM1
Inhibitory rec.
Activating rec.
CD56dim NK & cytotoxicity marker
Chemokines
CD56bright NK marker
CD56bright CD16- NK
BoL-specific CD56bright
CD56dim CD16+ NK
Average Expression
1.0
0.0
−1.0
Percent Expressed
25
50
75
100
E
NCAM1
FCGR3A
GZMB
GZMK
GNLY
KRT86
CXCR4
CXCR3
XCL1
XCL2
Max
0
Gene Expression
F
rBM Stamp
BoL
Patient 1
Patient 2
Patient 3
Patient 4
Patient 5
G
Myeloid cells
TREM2
FOLR2
HSPA6
IFIT3
IFIT1
CXCL10
TUBB
MKI67
CXCL12
APOC1
APOE
SELENOP
MRC1
CD163
ELANE
MPO
CDKN1C
RUNX3
FCGR3A
CD14
PTGS2
S100A12
S100A9
S100A8
FCER1A
CLEC10A
CLEC9A
IL3RA
CLEC4C
Proliferation marker
Macrophage marker
Progenitor marker
Monocyte marker
cDC marker
pDC marker
pDCs
cDC1
cDC2
Macrophages
Classical Monocytes
Imm. Monocytes
Myeloid prog.
NC Monocytes
Prolif. Monocytes
Rem. Monocytes
Average Expression
2
1
0
−1
Percent Expressed
25
50
75
100
H
CD14
S100A8
FCGR3A
CXCL8
C1QA
MRC1
APOE
CXCL12
CLEC9A
CLEC10A
Max
0
Gene Expression
I
rBM Stamp
BoL
Patient 1
Patient 2
Patient 3
Patient 4
Patient 5
J
cDC1/cDC2
Ratio
P = 0.06
rBM Stamp
BoL
NC/Classical Monocytes
log10(ratio)
P = 0.06
rBM Stamp
BoL
K
P01
P02
P03
P04
P05
TCR Fraction
rBM stamp
BoL
Non-pathogen TCR
Pathogen TCR
L
Cell Fraction
rBM stamp
BoL
P01
P02
P03
P04
P05
CD39+ PD1+
Cytotoxic
Effector
Memory
Early memory
Naïve
Remodelled
KLRD1+

Fig. S6. Heterogeneity of stromal, NK, myeloid and T cell subsets between breakout lesions and random bone marrow. A-B) Dotplot showing the expression of characteristic markers per stromal cell (A) or endothelial cell (B) subtypes. Color indicates the average expression level of the gene in the different subtypes, while the dot size specifies the percentage of cells with expression of the respective gene per subtype. ECM: extracellular matrix production. C) UMAP representation of stromal and endothelial cells per sample. Cells are coloured by subtype. D) Dotplot showing the expression of characteristic markers per NK cell subtype. Color indicates the average expression level of the gene in the different subtypes, while the dot size specifies the percentage of cells with expression of the respective gene per subtype. E) UMAP projection of NK cells colored by expression of exemplary NK cell subtype markers. F) UMAP representation of NK cells per sample. Cells are coloured by subtype. G) Dotplot showing the expression of characteristic markers per myeloid cell subtype. Color indicates the average expression level of the gene in the different subtypes, while the dot size specifies the percentage of cells with expression of the respective gene per subtype. pDC/cDC: plasmacytoid/conventional dendritic cells; Imm. monocytes: immature monocytes; Myeloid prog.: myeloid progenitors; NC monocytes: non-classical monocytes; Prolif. monocytes: proliferating monocytes; Rem. monocytes: remodeled monocytes. H) UMAP projection of all myeloid cells colored by expression of exemplary myeloid cell subtype markers. I) UMAP representation of myeloid cells per sample. Cells are colored by subtype. J) Cell fraction ratios between cDC1 and cDC2 subsets (left), and classical and non-classical monocytes between breakout lesion (BoL) and respective paired random bone marrow (rBM) stamps. Monocyte ratio is depicted in the log10 scale. Statistical analyses were performed using the Wilcoxon signed rank test, comparing BoL with paired rBM stamps. K) CD8 T cell fraction of all expanded T cell clones in paired BoL and rBM stamps, considering clones with a proportion of ≥1% and ≥10 cells in at least one of the paired samples. Pathogen-related TCR clones are highlighted in blue. L) Bar plots depicting relative cell fractions for the CD8 T cell subsets in BoL and paired rBM stamps.

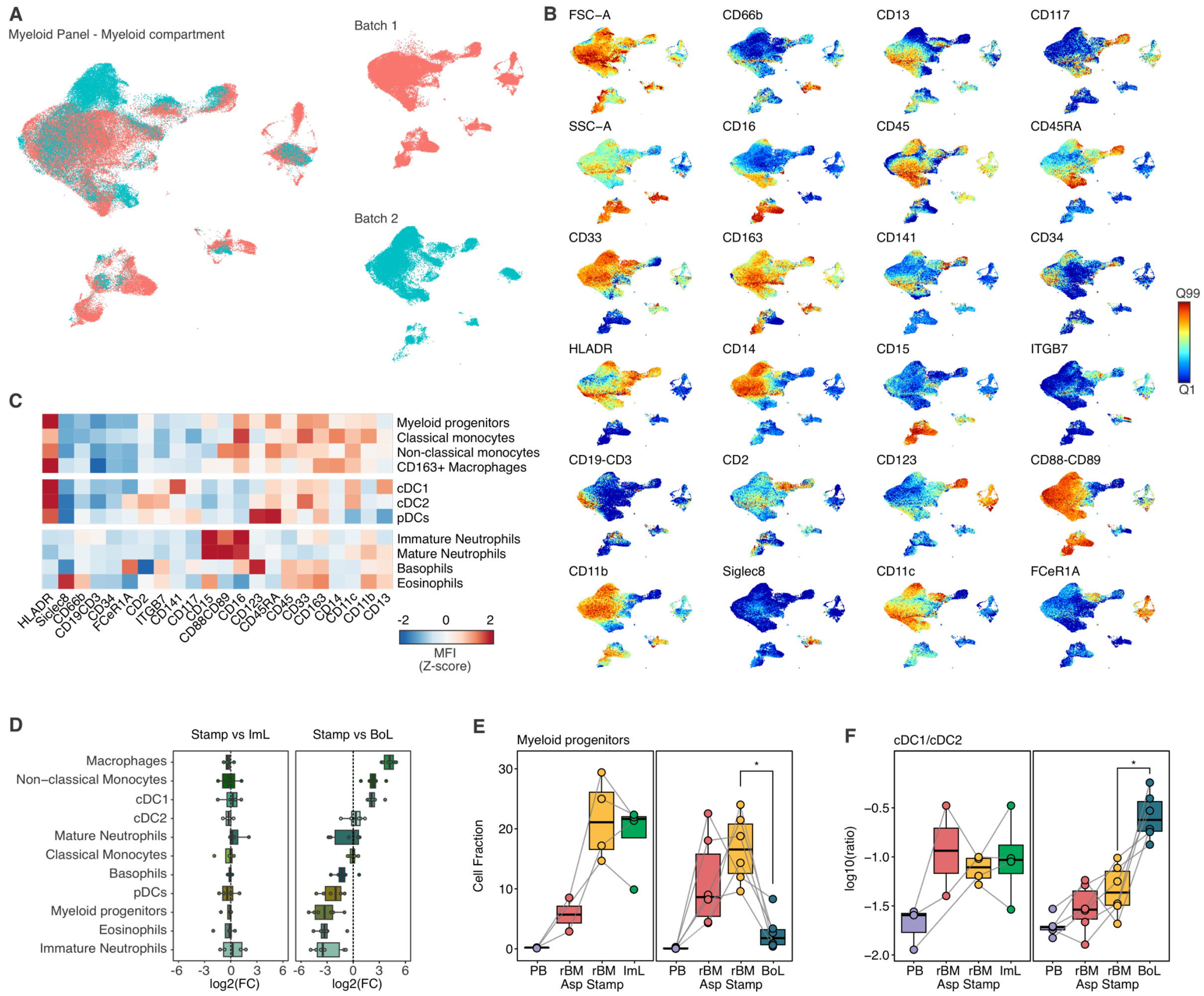

Fig. S7. Myeloid-focused cytometry panel. A) UMAP representation of the myeloid cell subsets obtained with the myeloid-focused panel, including cells from peripheral blood (PB), random bone marrow (rBM) aspirates and trephine biopsies (stamps) as well as intramedullary lesions (ImL) and breakout lesions (BoL). Cells are coloured per acquisition batch. B) Normalized expression of all markers included in the myeloid panel projected onto the UMAP representation. D) Heatmap displaying the scaled marker expression of the myeloid panel markers by cell type. D) Relative myeloid cell type abundance in ImL and BoL compared to paired rBM stamps. E) Cell fractions of myeloid progenitors across all sample types included in the study. F) Cell fraction ratios between cDC1 and cDC2 subsets across all sample types included in the study. Ratios are depicted in the log10 scale. Statistical analyses in (D-F) were performed using the Wilcoxon signed rank test. **P*<0.05, ***P*<0.01, ****P*<0.001, *****P*<0.0001.

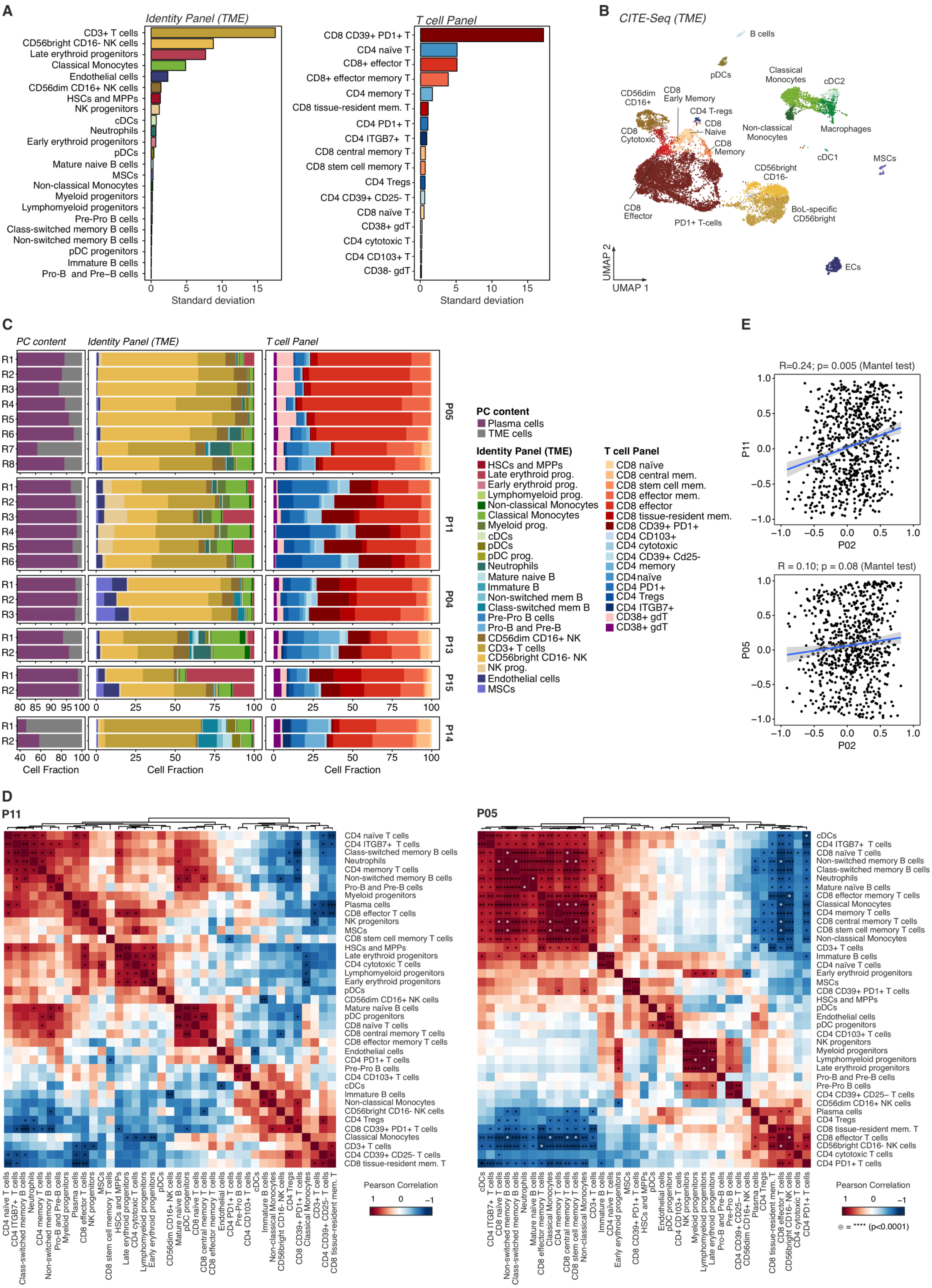
A
Identity Panel (TME)
CD3+ T cells
CD56bright CD16- NK cells
Late erythroid progenitors
Classical Monocytes
Endothelial cells
CD56dim CD16+ NK cells
HSCs and MPPs
NK progenitors
cDCs
Neutrophils
Early erythroid progenitors
pDCs
Mature naive B cells
MSCs
Non-classical Monocytes
Myeloid progenitors
Lymphomyeloid progenitors
Pre-Pro B cells
Class-switched memory B cells
Non-switched memory B cells
pDC progenitors
Immature B cells
Pro-B and Pre-B cells
Standard deviation
T cell Panel
CD8 CD39+ PD1+ T
CD4 naive T
CD8+ effector T
CD8+ effector memory T
CD4 memory T
CD8 tissue-resident mem. T
CD4 PD1+ T
CD4 ITGB7+ T
CD8 central memory T
CD8 stem cell memory T
CD4 Tregs
CD4 CD39+ CD25- T
CD8 naive T
CD38+ gdT
CD4 cytotoxic T
CD4 CD103+ T
CD38- gdT
Standard deviation
B
CITE-Seq (TME)
B cells
pDCs
Classical Monocytes
cDC2
CD56dim CD16+
CD8 Early Memory
CD4 T-regs
CD8 Naive
CD8 Cytotoxic
Non-classical Monocytes
Macrophages
CD8 Memory
cDC1
MSCs
CD56bright CD16-
CD8 Effector
PD1+ T-cells
BoL-specific CD56bright
ECs
UMAP 2
UMAP 1
C
PC content
Identity Panel (TME)
T cell Panel
P05
P11
P04
P13
P15
P14
Cell Fraction
PC content
Plasma cells
TME cells
Identity Panel (TME)
HSCs and MPPs
Late erythroid prog.
Early erythroid prog.
Lymphomyeloid prog.
Non-classical Monocytes
Classical Monocytes
Myeloid prog.
cDCs
pDCs
pDC prog.
Neutrophils
Mature naive B
Immature B
Non-switched mem B
Class-switched mem B
Pre-Pro B cells
Pro-B and Pre-B
CD56dim CD16+ NK
CD3+ T cells
CD56bright CD16- NK
NK prog.
Endothelial cells
MSCs
T cell Panel
CD8 naive
CD8 central mem.
CD8 stem cell mem.
CD8 effector mem.
CD8 effector
CD8 tissue-resident mem.
CD8 CD39+ PD1+
CD4 CD103+
CD4 cytotoxic
CD4 CD39+ Cd25-
CD4 memory
CD4naive
CD4 PD1+
CD4 Tregs
CD4 ITGB7+
CD38+ gdT
CD38+ gdT
E
R=0.24; p= 0.005 (Mantel test)
P11
P02
R = 0.10; p = 0.08 (Mantel test)
P05
P02
D
P11
P05
Pearson Correlation
= **** (p<0.0001)

Fig. S8. Spatially-resolved cellular ecotyping of breakout lesions. A) Standard deviation from the average cell fraction per cell type across the 16 regions in descending order for the identity (left) and T cell (right) flow cytometry panels. B) CITE-seq UMAP representation of all cells, excluding plasma cells, from 15/16 regions of patient P2. Cells are colored by celltype. C) Bar plots depicting cellular fractions obtained using the identity panel (Left: plasma cell count; Middle: TME cell fractions) as well as the T cell panel (Right) per region for six breakout lesions dissected into multiple regions. D) Correlation matrix of cell fractions obtained from flow cytometry data for patient P11 (left) and patient P05 (right). Plasma cell counts, relative fractions for tumor microenvironment (TME) cell types (identity panel) as well as relative fractions from CD4 and CD8 T cell subsets (T cell panel) were used. Color represents Pearson's correlation coefficient values. Statistically significant correlations are highlighted: *$P<0.05$, **$P<0.01$, ***$P<0.001$, •$P<0.0001$. E) Correlation between R values obtained from the correlation matrices of P11, P05 (B) and P02 (Fig. 4E) depicted as scatter plots. Each dot represents a correlation between two cell types. Statistical analysis was performed using a Mantel test in order to compare each matrix from B to the correlation matrix of P02 (Fig. 4E).

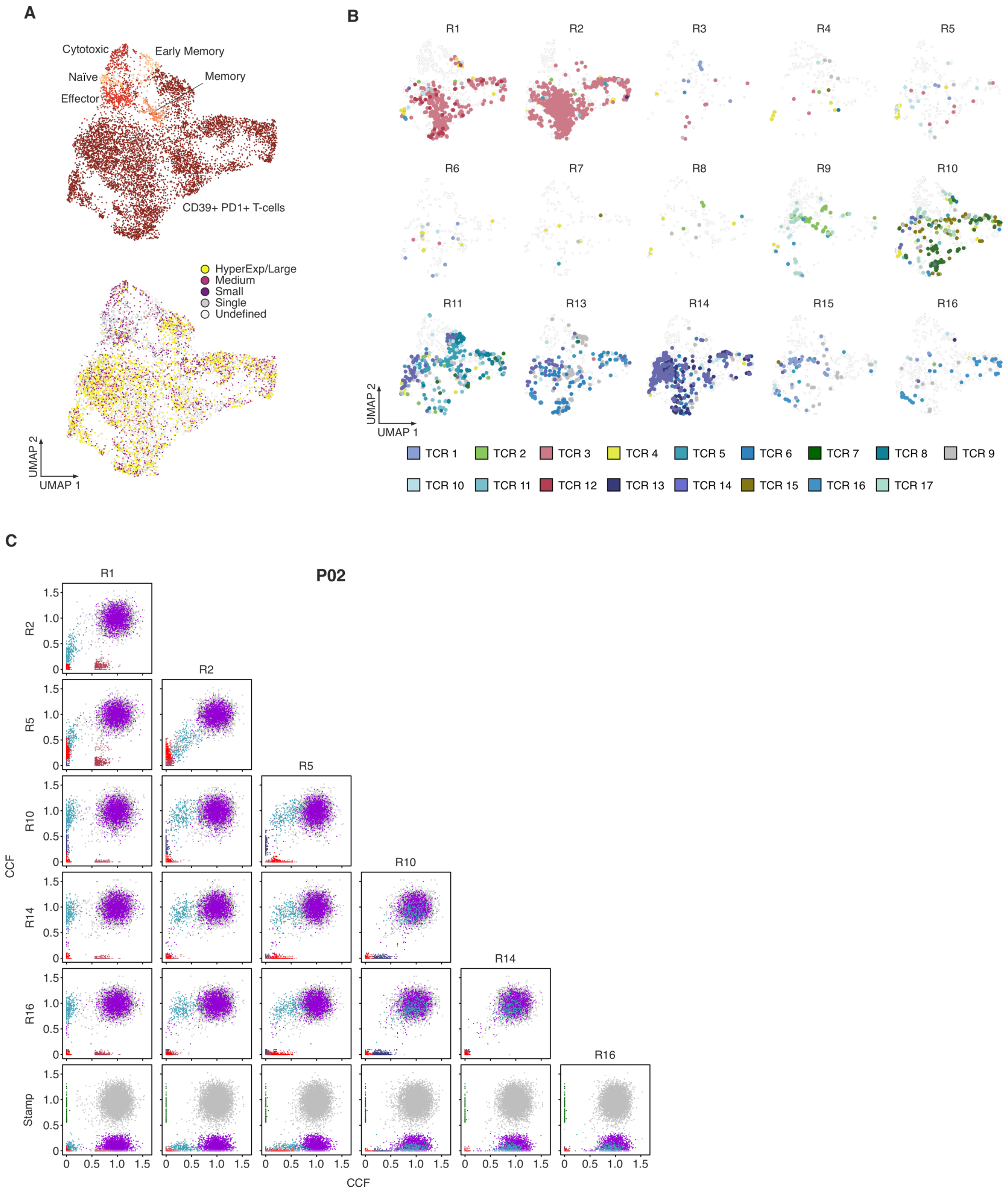

Fig. S9. Spatially-resolved subclonal architecture in breakout lesion P02. A) CITE-seq UMAP representation of CD8 T cells from 15/16 regions of the breakout lesion (BoL) from patient P02. Cells are coloured by celltype (top) and T cell expansion (down). HyperExp: hyperexpanded. B) UMAP representation of the CD8 T cells of the BoL from patient P02. Cells are coloured according to the TCR clone in each region. C) Whole genome sequencing (WGS) cancer clonal fraction (CCF) plots for total (synonymous and nonsynonymous) single nucleotide variants for 6 selected subregions from the breakout lesion of patient P02 and the matched random bone marrow stamp.

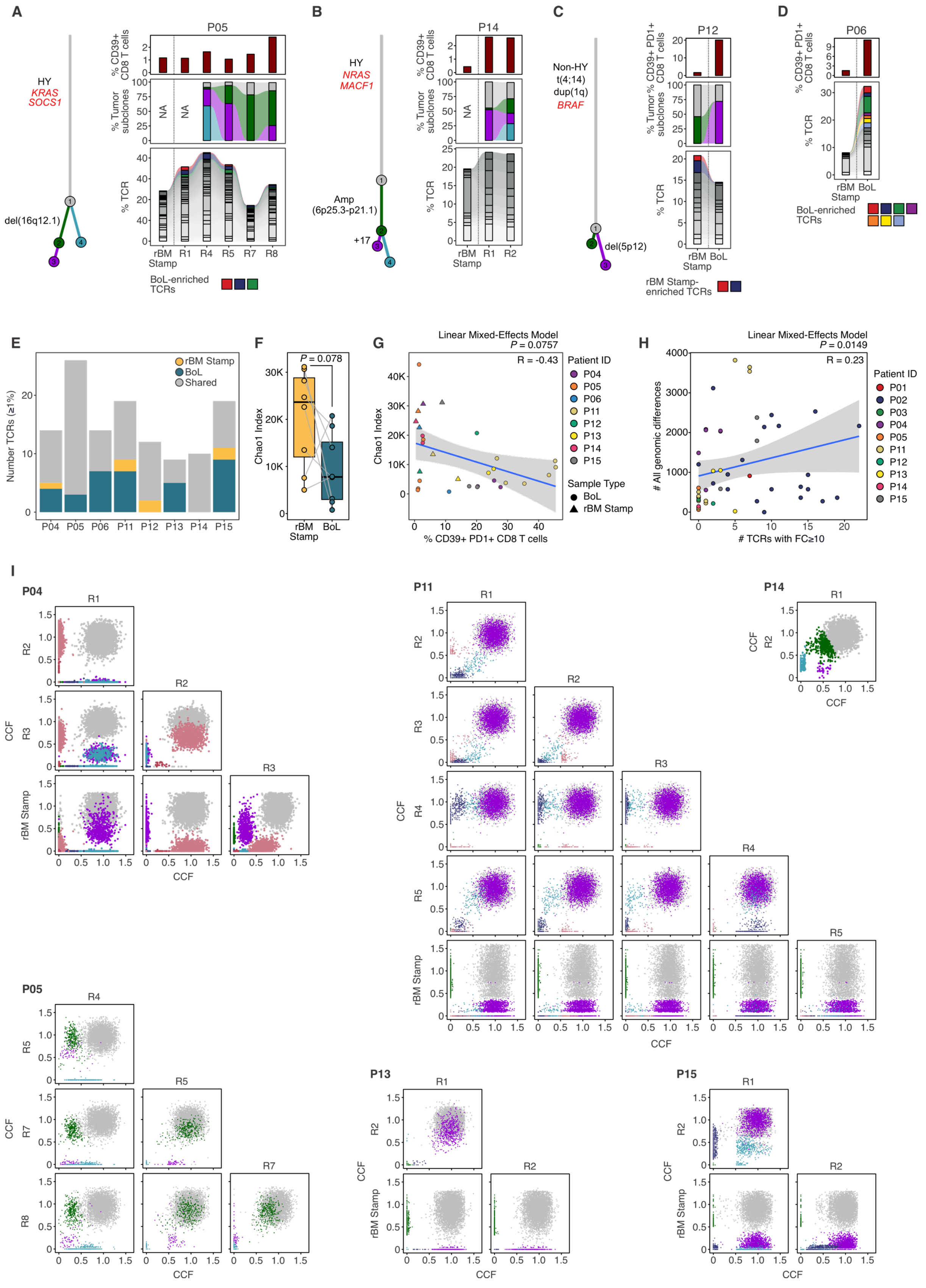
A
P05
HY
KRAS
SOCS1
del(16q12.1)
% CD39+ CD8 T cells
% Tumor subclones
% TCR
NA
rBM Stamp
R1
R4
R5
R7
R8
BoL-enriched TCRs
B
P14
HY
NRAS
MACF1
Amp (6p25.3-p21.1)
+17
R2
C
P12
Non-HY
t(4;14)
dup(1q)
BRAF
del(5p12)
% CD39+ PD1+ CD8 T cells
BoL
rBM Stamp-enriched TCRs
D
P06
E
rBM Stamp
Shared
Number TCRs (≥1%)
P04
P13
P15
P11
F
P = 0.078
Chao1 Index
G
Linear Mixed-Effects Model
P = 0.0757
R = -0.43
Patient ID
Sample Type
H
P = 0.0149
R = 0.23
# All genomic differences
# TCRs with FC≥10
P01
P02
P03
I
CCF
R3

Fig. S10. Spatially-resolved subclonal architecture and T cell receptor dynamics of additional breakout lesions. A-D) Summary plots of the bulk whole genome sequencing (WGS) and T cell receptor (TCR) sequencing experiments for paired breakout lesions (BoL) and random bone marrow (rBM) stamps. **Left**: phylogenetic tree. Selected mutations and copy number aberrations are highlighted in red and black, respectively. For patient P06, WGS was not available. HY: hyperdiploid karyotype; del: deletion; dup: duplication. Upper right: Barplots depicting the relative cell fraction of CD39+ PD1+ CD8 T cells per sample as determined by flow cytometry. Middle right: Alluvial plot depicting the fraction of tumor subclones per region as determined by WGS. Bottom right: Alluvial plot depicting cell fractions of expanded TCR clones (≥1% of total TCR repertoire) per region. Shared TCRs are shown in shades of gray, while TCR clones enriched in BoL or the rBM stamp (fold change ≥10) are depicted in color. E) Total number of expanded T cell clones (proportion ≥1% in at least one sample of pairs) that were shared between BoL and rBM stamp (gray), or unique to either rBM stamp (yellow) or BoL (blue) based on bulk TCR data. Only T cell clones with a fold change (FC) ≥10 in rBM stamp or in at least one BoL region were considered as enriched. F) T cell diversity according to the Chao1 index for breakout lesions (BoL) and paired random bone marrow (rBM) stamps for the bulk TCR-seq dataset. For patients with multiple breakout lesions, the average Chao1 index across all regions for each patient is shown. The boxplots show the median and the interquartile range, while the upper and lower whiskers show the highest and lowest value (excluding outliers), respectively. G) Correlation between Chao1 index and CD39+ PD1+ CD8 T cell fraction for patients with bulk TCR-seq data. Statistics were calculated using a linear mixed-effects model with patient id as a random effect. H) Correlation plot between number of expanded TCR clones (FC ≥10) and number of mutations defining subclones that were undetectable in one of the paired samples or showed a more than 3-fold difference in the median cancer clonal fraction (CCF) between the paired samples. scTCR-seq data was used for patient P01-P03 and bulk TCR-seq data was used for the other patients. Each dot represents a unique paired comparison. Statistics were calculated using a linear mixed-effects model with patients and methods as random effects. I) Whole genome sequencing cancer clonal fraction (CCF) plots for total (synonymous and nonsynonymous) single nucleotide variants for all patients with multiple subregions of a breakout lesion (P04, P05, P11 and P13-15).

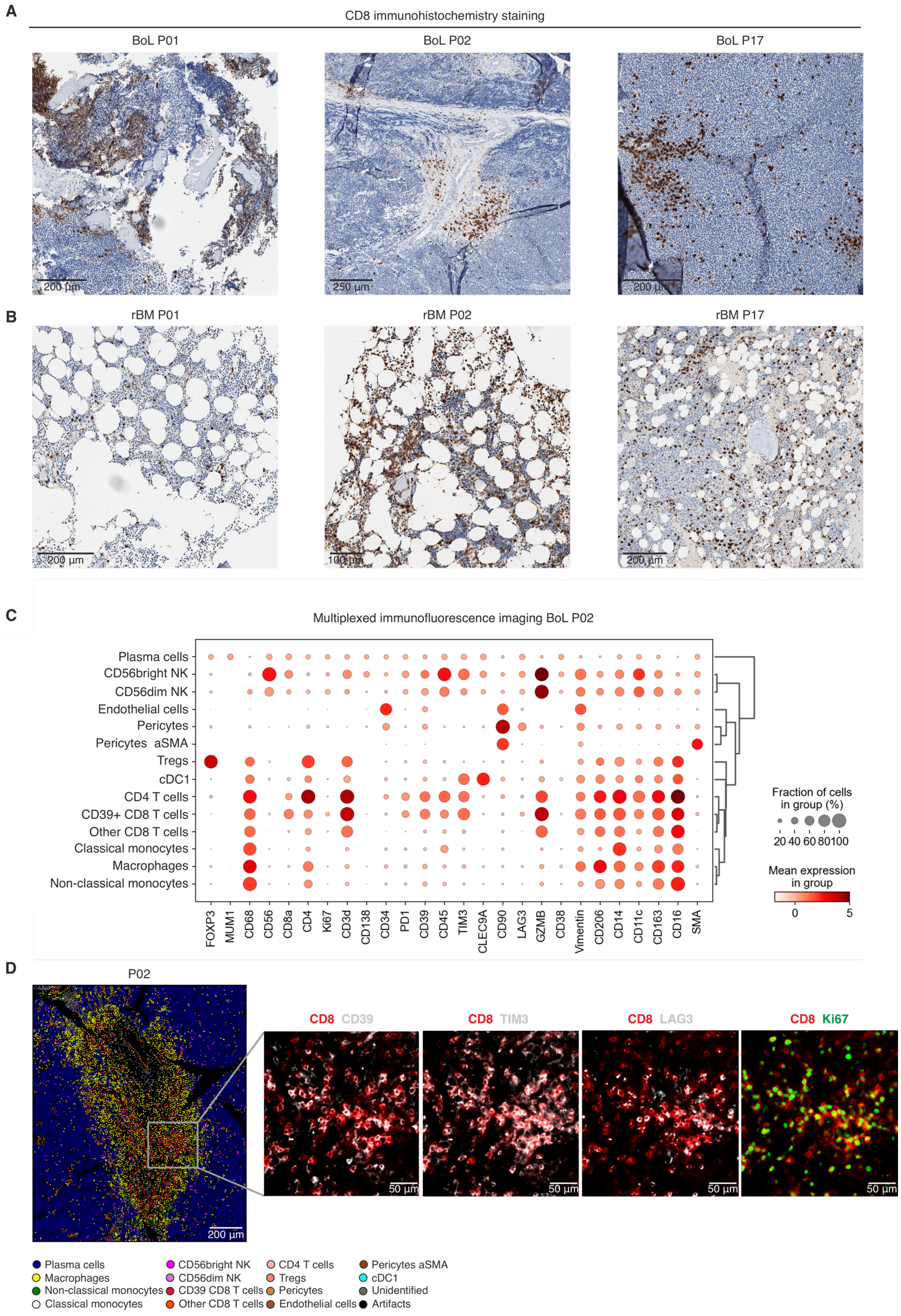
A
CD8 immunohistochemistry staining
BoL P01
BoL P02
BoL P17
200 µm
250 µm
B
rBM P01
rBM P02
rBM P17
200 µm
100 µm
200 µm
C
Multiplexed immunofluorescence imaging BoL P02
Plasma cells
CD56bright NK
CD56dim NK
Endothelial cells
Pericytes
Pericytes aSMA
Tregs
cDC1
CD4 T cells
CD39+ CD8 T cells
Other CD8 T cells
Classical monocytes
Macrophages
Non-classical monocytes
FOXP3
MUM1
CD68
CD56
CD8a
CD4
Ki67
CD3d
CD138
CD34
PD1
CD39
CD45
TIM3
CLEC9A
CD90
LAG3
GZMB
CD38
Vimentin
CD206
CD14
CD11c
CD163
CD16
SMA
Fraction of cells in group (%)
20 40 60 80100
Mean expression in group
0
5
D
P02
200 µm
CD8 CD39
CD8 TIM3
CD8 LAG3
CD8 Ki67
50 µm
Plasma cells
Macrophages
Non-classical monocytes
Classical monocytes
CD56bright NK
CD56dim NK
CD39 CD8 T cells
Other CD8 T cells
CD4 T cells
Tregs
Pericytes
Endothelial cells
Pericytes aSMA
cDC1
Unidentified
Artifacts

Fig. S11. Immunohistochemistry and multiplex immunofluorescence of breakout lesions. A) Examples of nodular T cell accumulations (immune islands) in breakout lesions of patients P01, P02 and P17 identified based on CD8 immunohistochemistry staining. B) Corresponding iliac crest sample of the patients in (A). C) Dotplot showing the expression of protein markers per cell subtype in multiplex immunofluorescence of a breakout lesion of patient P02. Color indicates the average expression level of the protein in the different subtypes, while the dot size specifies the percentage of cells with expression of the respective protein per subtype. D) Overlay images of the exhaustion markers CD39, LAG3 and TIM3 in CD8 T cells as well as the proliferation marker Ki67 within the immune island of the breakout lesion of patient P02.

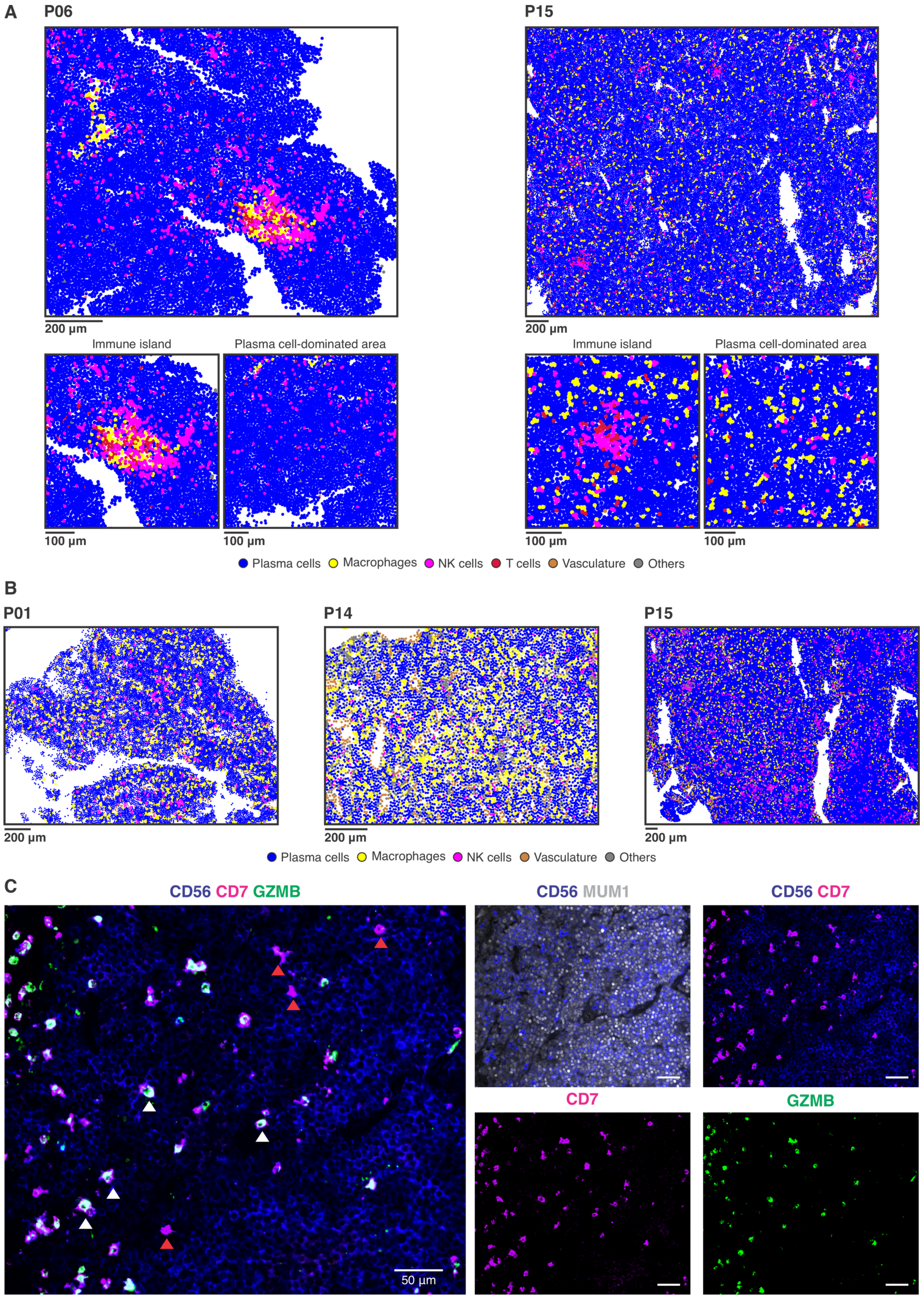
A
P06
200 µm
Immune island
Plasma cell-dominated area
100 µm
100 µm
P15
200 µm
Immune island
Plasma cell-dominated area
100 µm
100 µm
Plasma cells
Macrophages
NK cells
T cells
Vasculature
Others
B
P01
200 µm
P14
200 µm
P15
200 µm
Plasma cells
Macrophages
NK cells
Vasculature
Others
C
CD56 CD7 GZMB
50 µm
CD56 MUM1
CD56 CD7
CD7
GZMB

Fig. S12. Multiplex immunofluorescence of additional breakout lesions. A) Multiplex imaging of the breakout lesions from patients P06 and P15 as examples of lesions with immune islands. The zoom-ins highlight the immune island and a representative region of the surrounding plasma cell-dominated area. B) Exemplary multiplex images of the breakout lesions from patients P01, P14 and P15 showing tumor-infiltrating NK cells and macrophages. C) Overlay images for the breakout lesion of patient P02, including images for CD56, MUM1, CD7 and GZMB. CD7+CD56++GZMB+ breakout lesion (BoL)-specific NK cells are marked with white arrows, while conventional CD56bright NK cells are marked with red arrows.

Table S1: FACS "identity" panel

Antibody	Conjugate	Clone	Dilution	Source	Cat#	RRID
Mouse anti-human CD16	BUV395	3G8	1:100	BD Biosciences	563785	AB_2744293
Mouse anti-human CD138	BUV496	MI15	1:50	BD Biosciences	749874	AB_2874114
Mouse anti-human CD19	BUV563	SJ25C1	1:100	BD Biosciences	612916	AB_2870201
Mouse anti-human CD11b	BUV615	M1/70	1:50	BD Biosciences	751140	AB_2875166
Mouse anti-human CD27	BUV661	M-T271	1:50	BD Biosciences	741609	AB_2871017
Mouse anti-human CD124	BUV737	hIL4R-M57	1:50	BD Biosciences	749111	AB_2873503
Mouse anti-human CD45	BUV805	HI30	1:100	BD Biosciences	612891	AB_2870179
Mouse anti-human CD10	BV421	HI10a	1:50	BioLegend	312218	AB_2561833
eFluor™ 506	BV480		1:1000	Life Technologies	65-0866-18	N/A
Mouse anti-human CD90	BV605	5E10	1:50	BD Biosciences	747750	AB_2872219
Mouse anti-human CD335	BV650	NKp46	1:50	BD Biosciences	563230	AB_2872219
Mouse anti-human CD3	BV711	SK7	1:100	BD Biosciences	740832	AB_2740489
Mouse anti-human IgD	BV750	IA6-2	1:100	BD Biosciences	747484	AB_2868411
Mouse anti-human CD146	BV786	P1H12	1:50	BioLegend	361030	AB_2800996
Mouse anti-human CD45RA	BB515	HI100	1:100	BD Biosciences	564552	AB_2738841
Mouse anti-human CD38	PerCPCy5.5	HIT2	1:100	BD Biosciences	551400	AB_394184
Mouse anti-human CD34	PE	581	1:5	BD Biosciences	555822	AB_396151
Mouse anti-human CD56	PE-CF594	5.1H11	1:50	BioLegend	362544	AB_2565922
Mouse anti-human ITGB7	PE-Cy5	FIB504	1:5	BD Biosciences	551059	AB_394026
Mouse anti-human CD271	PE-Cy7	ME20.4-1.H4	1:50	Miltenyi	130-113-422	AB_2733220
Mouse anti-human CD33	APC	WM53	1:50	BD Biosciences	551378	AB_398502
Mouse anti-human CD71	AF700	M-A712	1:100	BD Biosciences	563769	AB_2738415
Mouse anti-human HLA-DR	APC Cy7	L243	1:100	BioLegend	307618	AB_493586

Table S2: FACS T cell focused panel

Antibody	Conjugate	Clone	Dilution	Source	Cat#	RRID
Mouse anti-human CD4	BUV395	SK3	1:100	BD Biosciences	563550	AB_2738273
Mouse anti-human CD8	BUV496	SK1	1:100	BD Biosciences	741199	AB_2870759
Mouse anti-human CD19/CD71/CD33	BUV563	SJ25C1/L01.1/WM53	1:100	BD Biosciences	612916/748314/ 741369	AB_287020/ AB_2872735/AB_2870869
Mouse anti-human CD314	BUV615	1D11	1:50	BD Biosciences	751232	AB_2875253
Mouse anti-human CXCR3	BUV661	1C6/CXCR3	1:50	BD Biosciences	741649	AB_2871046
Mouse anti-human CD38	BUV737	HIT2	1:50	BD Biosciences	741837	AB_2871172
Mouse anti-human CD45RO	BUV805	UCHL1	1:100	BD Biosciences	748367	AB_2872786
Mouse anti-human CD28	BV421	L293	1:100	BD Biosciences	742525	AB_2740843
eFluor™ 506	BV480		1:1000	Life Technologies	65-0866-18	N/A
Mouse anti-human CD103	BV605	Ber-ACT8	1:50	BD Biosciences	743652	AB_2741654
Mouse anti-human CD279	BV650	EH12.1	1:50	BD Biosciences	564104	AB_2738595
Mouse anti-human CD94	BV711	HP-3D9	1:50	BD Biosciences	743952	AB_2741874
Mouse anti-human TCRab	BV750	IP26	1:50	BD Biosciences	747180	AB_2871914
Mouse anti-human CD95	BV786	DX2	1:50	BD Biosciences	740991	AB_2740614
Mouse anti-human CD45RA	BB515	HI100	1:100	BD Biosciences	564552	AB_2738841
Mouse anti-human CD69	PerCPCy5.5	FN50	1:50	BD Biosciences	560738	AB_1727510
Mouse anti-human CD39	PE	A1	1:50	BioLegend	328208	AB_940429
Mouse anti-human CD25	PE-CF594	M-A251	1:50	BD Biosciences	562403	AB_11151919
Mouse anti-human ITGB7	PE-Cy5	FIB504	1:5	BD Biosciences	551059	AB_394026
Mouse anti-human TCRgd	PE-Cy7	11F2	1:20	BD Biosciences	655410	AB_2870377
Mouse anti-human CD197	APC	2-L1-A	1:50	BD Biosciences	566762	AB_2869854
Mouse anti-human CD127	AF700	HIL-7R-M21	1:50	BD Biosciences	565185	AB_2739099

Mouse anti-human CD3	APC-Cy7	SK7	1:20	BD Biosciences	641415	AB_2870309

Table S3: FACS antibodies used for sorting of flow-enriched cell types (CITE-seq workflow)

Antibody	Conjugate	Clone	Dilution	Source	Cat#	RRID
Mouse anti-human CD3	BUV395	UCHT1	1:50	BD Biosciences	563546	AB_2744387
Mouse anti-human CD19	BUV563	SJ25C1	1:100	BD Biosciences	612916	AB_2870201
Mouse anti-human CD45	BUV805	HI30	1:100	BD Biosciences	612891	AB_2870179
Mouse anti-human CD146	BV421	P1H12	1:50	BD Biosciences	564325	AB_2738747
eFluor™ 506	BV480		1:1000	Life Technolgies	65-0866-18	N/A
Mouse anti-human CD90	BV605	5E10	1:50	BD Biosciences	747750	AB_2872219
Calcein AM Viability Dye (UltraPure Grade)	BB515			Life Technologies	65-0853-78	N/A
Mouse anti-human CD38	PerCPCy5.5	HIT2	1:100	BD Biosciences	551400	AB_394184
Mouse anti-human CD34	PE	581	1:30	BD Biosciences	555822	AB_396151
Mouse anti-human CD271	PE-Cy7	ME20.4-1.H4	1:100	Miltenyi	130-113-422	AB_2733220
Mouse anti-human CD7	APC	M-T701	1:50	BD Biosciences	561604	AB_10893354
Mouse anti-human HLA-DR	APC Cy7	L243	1:100	BioLegend	307618	AB_493586

Table S4: FACS Myeloid-focused panel

Antibody	Conjugate	Clone	Dilution	Source	Cat#	RRID
Mouse anti-human CD16	BUV395	3G8	1:100	BD Biosciences	563785	AB_2744293
Mouse anti-human CD163	BUV496	GHI/61	1:50	BD Biosciences	750581	AB_2874716
Mouse anti-human CD14	BUV563	MφP9	1:100	BD Biosciences	741441	AB_2870917
Mouse anti-human CD2	BUV615	RPA-2.10	1:100	BD Biosciences	751345	AB_2875353
Mouse anti-human Siglec8	BUV661	837535	1:50	BD Biosciences	750239	AB_2874437
Mouse anti-human CD13	BUV737	WM15	1:50	BD Biosciences	741828	AB_2871163
Mouse anti-human CD45	BUV805	HI30	1:100	BD Biosciences	612891	AB_2870179
Mouse anti-human CD141	BV421	1A4	1:50	BD Biosciences	565321	AB_2739180
eFluor™ 506	BV480		1:1000	Life Technologies	65-0866-18	N/A
Mouse anti-human CD15	BV605	W6D3	1:50	BioLegend	323032	AB_2562132
Mouse anti-human CD123	BV650	9F5	1:50	BD Biosciences	740588	AB_2740289
Mouse anti-human CD11c	BV711	B-ly6	1:100	BD Biosciences	563130	AB_2738019
Mouse anti-human CD117	BV750-P	104D2	1:100	BD Biosciences	747514	AB_2872179
Mouse anti-human CD45RA	BV786	HI100	1:100	BD Biosciences	563870	AB_2738459
Mouse anti-human CD11b	FITC	M1/70	1:50	BioLegend	101206	AB_312789
Mouse anti-human CD66b	PerCPCy5.5	G10F5	1:30	BioLegend	305108	AB_2077855
Mouse anti-human CD34	PE	8G12	1:5	BD Biosciences	555822	AB_396151
Mouse anti-human FceR1A	PE-CF594	AER37	1:50	BioLegend	334634	AB_2571906
Mouse anti-human ITGB7	PE-Cy5	FIB504	1:5	BD Biosciences	551059	AB_394026
Mouse anti-human CD88/89	PE-Cy7	S5/1 /A59	1:50	BioLegend	344308/354108	AB_11126750/AB_2565259
Mouse anti-human CD33	APC	WM53	1:50	BD Biosciences	551378	AB_398502
Mouse anti-human CD19/CD3	AF700	SJ25C1/ SK7	1:50	BioLegend	363034/ 344822	AB_2616936/AB_2563420
Mouse anti-human HLA-DR	APC Cy7	L243	1:100	BioLegend	307618	AB_493586

Table S5: Multiplexed imaging panel used for the COMET^{TM} platform

Antibody	Host	Conjugate	Clone	Dilution	Source	Cat#	RRID
FOXP3	Rabbit	None	SP97	1:100	Invitrogen,	MA5-16365	AB_2537884
MUM1	Mouse	None	Mum1-p	1:100	Agilent Technologies	M725901-2	N/A
CD68	Mouse	None	KP1	1:80	Life Technologies	14068882	AB_11151139
CD56	Rabbit	None	MRQ-42	1:100	Origene	TA327646	N/A
PAX5	Rabbit	None	D7H5X	1:100	Cell Signaling	93009SF	N/A
CD8a	Mouse	None	AMC908	1:50	Life Technologies	14000882	AB_2572848
CD4	Rabbit	None	EPR6588	1:80	Abcam	ab133616	AB_2750883
Ki67	Mouse	None	B56	1:50	BD Biosciences	556003	AB_396287
CD3d	Rabbit	None	SP162	1:80	Sigma-Aldrich	SAB5500057-100UL	N/A
CD138	Mouse	None	B-A38	1:100	Bio-RAD	MCA2459GA	AB_566508
CD34	Mouse	None	QBEnd/10+HPCA 1/763	1:75	Biomol	NSJ-V2999-100UG	N/A
PD1	Rabbit	None	EPR4877(2)	1:80	Abcam	ab137132	AB_2894867
CD39	Rabbit	None	EPR20627	1:200	Abcam	ab236038	AB_2943150
CD45	Mouse	None	2B11+PD7/26	1:100	BIO-TECHNE	NBP2-34287-0.1mg	N/A
CD45RA	Mouse	None	HI100	1:50	Biolegend	304102	AB_2621351
CD366/TIM3	Rabbit	None	D5D5R	1:80	Cell Signaling	45208S	N/A
CLEC9A	Rabbit	None	EPR22324	1:200	Abcam	ab245121	N/A
CD90	Rabbit	None	EPR3132	1:100	Abcam	ab181885	N/A
LAG3	Rabbit	None	EPR20261	1:100	Abcam	ab209236	AB_2883982
GZMB	Rabbit	None	D6E9W	1:100	Cell Signaling	79903SF	N/A
CD38	Rabbit	AF555	PR4106	1:200	Abcam	ab279332	N/A
Vimentin	Rabbit	AF647	D21H3	1:300	Cell Signaling	9856S	AB_10834530
CD206	Rabbit	None	E2L9N	1:100	Cell Signaling	91992S	AB_2800175
CD14	Rabbit	None	D7A2T	1:100	Cell Signaling	75181S	AB_2799865
CD11c	Rabbit	AF647	EP1347Y	1:100	Abcam	ab275338	N/A
CD163	Rabbit	None	EPR14643	1:100	Abcam	ab188571	N/A
CD16	Rabbit	AF555	EPR16784	1:50	Abcam	ab281764	AB_3532177
aSMA	Mouse	AF647	1A4	1:200	Thermo Fischer	50976082	AB_2574362

PAX5	Rabbit	None	D7H5X	1:100	Cell Signaling	93009SF	AB_3080867

Data file S1: Patient characteristics

Data file S2: Sample overview and readouts

Data file S3: Patient estimates and proportions of the flow cytometry and CITE-seq dataset

Data file S4: T cell receptor information of patients P01-P05

Data file S5: Patient estimates and proportions of all patients with multiple regions

Data file S6: T cell receptor information of 15/16 regions from breakout lesion, rBM and PB sample from patient P02.

Data file S7: Source data table for all figures.
